# Effect Size and Replicability in Genetic Studies of Athletic Performance: A Meta-Analytical Review

**DOI:** 10.3390/genes16091040

**Published:** 2025-08-31

**Authors:** Kinga Wiktoria Łosińska, Paweł Cięszczyk, Giovanna Ghiani, Adam Maszczyk

**Affiliations:** 1 Department of Molecular Biology, Gdansk University of Physical Education and Sport, Kazimierza Górskiego 1, 80-336 Gdansk, Poland; pawel.cieszczyk@awf.gda.pl; 2 Sports Physiology Laboratory, University of Cagliari, Università 40, 09124 Cagliari, Italy; giovannam.ghiani@unica.it; 3 Department of Sports Theory and Practice, Jerzy Kukuczka Academy of Physical Education in Katowice, Mikolowska 72a, 40-065 Katowice, Poland; a.maszczyk@awf.katowice.pl

**Keywords:** sports genetics, effect size, ACTN3, *ACE*, meta-analysis, replication, athletic performance

## Abstract

Background/Objectives: This meta-analytical review assesses the relationship between effect size and replication success in genetic studies of athletic performance, focusing on the *ACTN3* and *ACE* polymorphisms across power- and endurance-based sports. The analysis revealed substantial heterogeneity in reported effect sizes (overall I^2^ = 72.3%), indicating considerable variability between studies, likely influenced by differences in population genetics, study design, and sample size. Methods: For *ACTN3*, the pooled effect sizes were 1.40 (95% CI: 1.18–1.65) for power sports and 1.35 (95% CI: 1.12–1.58) for endurance sports. Although the difference between these estimates is small, it reached statistical significance (*p* = 0.0237), reflecting the large sample size, but it remains of limited practical and clinical significance. For the *ACE* polymorphism, effect sizes were similar in both endurance (ES = 1.22, 95% CI: 1.05–1.41) and power sports (ES = 1.20, 95% CI: 1.03–1.43), with overlapping confidence intervals, indicating no meaningful difference in association strength between sport types. Effect sizes were calculated as odds ratios (OR) with 95% confidence intervals for case–control designs, with standardized conversion protocols applied for alternative study designs reporting standardized mean differences or regression coefficients. Results: Publication bias was detected, particularly in smaller studies on *ACTN3* and power sports (Egger’s test *p* = 0.007). The pooled effect of *ACTN3* in power sports (OR 1.40, 95% CI: 1.18–1.65, 95% PI: 0.89–2.20) was adjusted to OR 1.32 (95% CI: 1.15–1.51) following trim-and-fill publication bias correction. The high degree of heterogeneity (I^2^ = 72.3%) cautions against overgeneralization of the pooled results and highlights the need for careful interpretation, robust replication studies, and standardized methodologies. Conclusions: The findings emphasize that, while genetic markers such as *ACTN3* and *ACE* are statistically associated with athletic performance, the magnitude of these associations is modest and should be interpreted conservatively. Methodological differences and publication bias continue to limit the reliability of the evidence. Future research should prioritize large, well-powered, and methodologically consistent studies—ideally genome-wide approaches—to better account for the polygenic and multifactorial nature of elite athletic ability.

## 1. Introduction

Athletic performance is a complex trait, influenced by genetic, environmental, physiological, and psychological factors. Recent advances in genomics have enabled researchers to identify specific genetic variants that may contribute to variability in strength, speed, endurance, and injury susceptibility in both elite and recreational athletes.

*ACTN3* and *ACE* are the two most robust genetic markers linked to sports performance. The *ACTN3* R577X polymorphism determines the presence or absence of the alpha-actinin-3 protein in fast-twitch muscle fibers, influencing muscle power and sprint ability. Athletes with the RR or RX genotype more frequently excel in strength and speed-oriented disciplines, while the XX genotype is overrepresented among endurance athletes. The *ACE* I/D polymorphism affects the regulation of the renin–angiotensin pathway, thus modulating cardiovascular efficiency and blood pressure—features critical for both power and endurance sports. Both markers have been repeatedly associated with measurable differences in athletic achievement, rendering them exemplary candidate genes for meta-analytical and replication-focused research in exercise genomics.

Besides *ACTN3* and *ACE*, recent meta-analyses and large-scale association studies have identified additional genetic polymorphisms, such as PPARGC1A (peroxisome proliferator-activated receptor gamma coactivator 1-alpha), BDNF (brain-derived neurotrophic factor), COL5A1 (collagen type V alpha 1 chain), AMPD1 (adenosine monophosphate deaminase 1), and VEGF (vascular endothelial growth factor), which contribute modestly to athletic capability and injury risk. Nevertheless, the effect sizes observed for these genes are generally small and less consistently replicated than those found for *ACTN3* and *ACE*.

The challenge of reproducibility in sports genetics emerges not merely as an echo of broader issues in genetic association studies, but as a problem that acquires unique dimensions within this domain. Although numerous studies report statistically significant associations between particular genetic polymorphisms, such as *ACTN3* and *ACE*, and athletic performance, the replicability and substantive relevance of these findings remain deeply uncertain and often controversial [1,2,3,4]. Unlike other fields, sports genetics is characterized by significant heterogeneity of phenotypes, high selection pressure for extreme traits, and an overrepresentation of small, specialized cohorts, all of which can exacerbate instability and overestimation of research outcomes.

A central concern is the interpretation of reported effect sizes. While large effect sizes might suggest robust biological relevance, in the context of sports genetics they frequently reflect methodological artifacts, including small and non-representative samples, suboptimal phenotypic classification, and statistical or publication biases [5,6,7,8]. These factors not only inflate the perceived strength of genetic associations, but also impede meaningful replication in broader, more diverse athletic populations.

Of particular importance is the phenomenon known as the “winner’s curse”, wherein initial reports, often emerging from underpowered studies or unique samples, systematically overestimate true genetic effects [9,10,11]. In sports genetics, this overestimation is magnified by the tendency to publish positive, striking findings and by the practical difficulties in recruiting large, independent cohorts of elite athletes for validation. As a result, meta-analytical syntheses attempting to clarify the magnitude and generalizability of these effects are themselves limited by the quality and scope of primary research.

Further complicating the landscape are discrepancies in study populations, variations in training history, and environmental confounders intrinsic to sports disciplines [12,13]. High variability in phenotype definition and measurement, coupled with population-specific allele frequencies, contributes to inconsistent replication of genetic associations. Even in meta-analyses, the accumulation of heterogeneous datasets does not necessarily resolve these sources of error; rather, it may propagate or mask underlying methodological and publication biases.

In this context, several interdependent factors determine the reliability and replicability of genetic findings in sports performance research. Larger sample sizes generally yield more accurate effect size estimates and enhance the likelihood of successful replication, while studies with insufficient power are prone to reporting exaggerated associations that seldom persist in subsequent investigations. The influence of genetic background, training regimen, and ethnicity further muddles attempts to generalize results across populations. In addition, methodological stringency, including the use of independent replication cohorts, transparent reporting, and comprehensive statistical analysis, remains essential yet variably implemented. Finally, the pervasive impact of selective reporting and publication bias distorts the body of evidence, as studies with statistically significant or large effect sizes are disproportionately published, leading to systemic overestimation of genetic contributions [6,14,15].

This meta-analytical review directly addresses these complexities by systematically evaluating the relationship between effect size and replication success in genetic studies of athletic performance, with a specific focus on the *ACTN3* and *ACE* polymorphisms in power- and endurance-oriented sports. Through quantitative comparison of effect sizes and analysis of replication patterns in the literature, we aim to clarify not only the reliability of individual associations but also the methodological conditions under which robust and replicable genetic findings are most likely to emerge. Insight into these dynamics is essential for refining future study designs and advancing the practical and scientific utility of sports genomics.

## 2. Materials and Methods

### 2.1. Study Design and Protocol Registration

**Figure 1:**
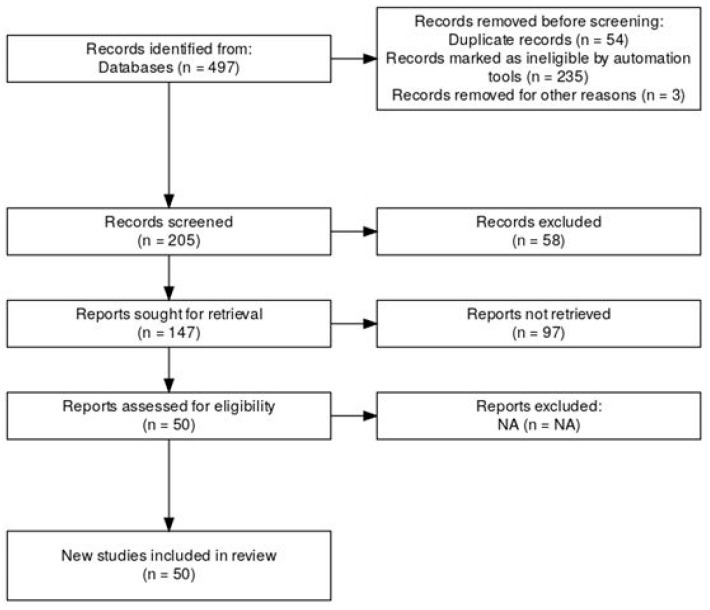
PRISMA flow diagram for effect size and replica ability in genetic studies of athletic performance.

This systematic review and meta-analysis was conducted following the Preferred Reporting Items for Systematic Reviews and Meta-Analyses (PRISMA 2020) guidelines. The review protocol was prospectively registered in the PROSPERO database, under the title “Effect Size and Replicability in Genetic Studies of Athletic Performance: A Meta-Analytical Review” (registration number: CRD420251019932). The PRISMA flow diagram illustrating the identification, screening, eligibility assessment, and inclusion of studies is presented in Figure 1.

### 2.2. Eligibility Criteria

Studies were included in this review based on the following criteria.

- Publication in peer-reviewed journals and focus on genetic markers related to athletic performance.
- Reporting of explicit, primary effect sizes (odds ratios, Cohen’s d, R-squared values), or providing full raw data (means, standard deviations, allele counts) enabling the rigorous and standardized calculation of effect sizes as detailed in the Data Synthesis section below.
- Clear description of the study design (case–control, cohort, or quantitative trait association).
- Examination of associations between genetic polymorphisms and discrete, well-defined sports performance outcomes (e.g., endurance performance, explosive strength, specific injury risk).
- Inclusion of human elite or trained athlete populations
- Full text in English, available for thorough data extraction.
- Exclusion criteria included.
- Did not directly report primary effect sizes or lacked sufficient raw data for reproducible effect size calculation; all effect size conversions were conducted following widely accepted methodological conventions to minimize error and preserve comparability, as specified below.
- Non-human genetic studies or in vitro research.
- Were conference abstracts, commentaries, editorials, or reviews lacking primary data.
- Studies with incomplete data that could not be retrieved from corresponding authors.

### 2.3. Clarification on Effect Size Calculation and Harmonization

In cases where effect size metrics were missing, standardized and validated formulas were used to convert available data (contingency tables, means, standard deviations) into odds ratios (OR) or standardized mean differences (SMD, Cohen’s d). These conversions were performed in accordance with guidelines for genetic meta-analyses, and all assumptions and formulas are documented in the Supplementary Materials File.

To ensure consistency and minimize heterogeneity introduced by differing metrics, we harmonized effect sizes by transforming all eligible outcomes to a uniform metric—primarily OR for binary traits and Cohen’s d for quantitative traits. Studies reporting only R^2^ or regression coefficients were included only if sufficient additional data were available to enable accurate conversion. Separate meta-analyses were conducted for case–control studies and for quantitative trait association studies, and subgroup and sensitivity analyses were performed to evaluate the impact of pooling different study designs and conversion methods.

### 2.4. Justification for Study Inclusion and Heterogeneity Controls

To prevent excessive heterogeneity arising from broad eligibility criteria, only studies with comparable outcome definitions, control group characteristics, and genotyping methods were included. Effect size harmonization protocols were implemented to maintain methodological consistency across diverse study designs. In the primary analyses, odds ratios (OR) were used as the standardized effect metric for case–control studies, calculated using the Mantel–Haenszel method. Studies reporting SMD (Cohen’s d) were converted to OR using the formula OR = exp(d × π/√3). Regression coefficients were transformed using the Chinn (2000) approximation: OR = exp(β × SD_exposure). Sensitivity analyses included only studies that originally reported OR to assess any bias introduced by conversions. Heterogeneity was preemptively quantified using I^2^ and Cochran’s Q statistics, followed by sensitivity analyses excluding studies with outlier methodologies or extreme heterogeneity indicators. Subgroup analyses by phenotype, study design, and population were also performed to clarify sources of variation and improve the interpretability of pooled effects.

### 2.5. Information Sources and Search Strategy

A comprehensive literature search was performed across PubMed, Web of Science, Scopus, and SPORTDiscus. The final update occurred in January 2025. The search combined subject headings and free-text terms on sports genetics, effect size reporting, and replication. Gray literature, preprint servers (bioRxiv, medRxiv), and institutional databases were scrutinized. The reference lists of relevant systematic reviews and primary studies were manually screened. The full search strategy, including all queries and date ranges, is provided in Supplementary Materials (Section S1). Publication bias was mitigated by inclusion of gray literature and outreach to field experts for unpublished datasets.

Search terms included: (“genetic polymorphisms” OR “sports genetics” OR “gene variants” OR “genetic association study” OR “genetic predisposition” OR “genome-wide association study” OR “SNPs” OR “athlete genetics” OR “sports genomics” OR “genetic markers for performance”) AND (“effect size” OR “odds ratio” OR “Cohen’s d” OR “heritability” OR “genetic contribution” OR “genetic risk score” OR “polygenic risk score” OR “allelic frequency impact” OR “genetic effect magnitude”) AND (“athletic performance” OR “endurance” OR “power” OR “speed” OR “strength” OR “sprint” OR “VO2 max” OR “muscle fiber composition” OR “explosive strength” OR “maximal oxygen uptake” OR “physical performance traits” OR “genotype-phenotype correlation”).

### 2.6. Study Selection and Data Extraction

A review protocol was developed prior to study initiation and is available upon request. From a total of 497 records identified, duplicates (*n* = 54) and ineligible records identified by automated algorithms (*n* = 235) were excluded. Title and abstract screening yielded 205 records; subsequently, 147 studies were fully assessed for eligibility. Ultimately, 50 studies met the stringent inclusion criteria and underwent full data extraction (Figure 1). The predominant reason for exclusion was the absence of reported or calculable effect sizes (*n* = 97).

Two independent reviewers conducted the screening process, with all discrepancies resolved through discussion or by a third reviewer. Inter-rater reliability was quantified using Cohen’s kappa statistic to ensure consistency.

Multiple comparison procedures were implemented to control family-wise error rates across subgroup analyses. Primary hypotheses examining *ACTN3* and *ACE* associations with power versus endurance phenotypes were pre-specified in the PROSPERO protocol and analyzed without α-adjustment. Secondary exploratory analyses including gene-by-sport interactions and population-stratified comparisons employed false discovery rate (FDR) control using the Benjamini–Hochberg procedure with q < 0.05. Meta-regression analyses investigating continuous moderators (study quality, publication year, sample size) were flagged as hypothesis-generating and interpreted with appropriate caution regarding Type I error inflation.

A fully standardized data extraction form was employed and included detailed, pre-defined fields for:

- Authors, publication year, sample size, and precise study design (including specification of case–control or quantitative trait association).
- Comprehensive documentation of all genetic polymorphisms analyzed, genotyping methods, and allele frequencies.
- Explicit outcome variables with operational definitions and detailed measurement protocols, ensuring harmonization of athletic performance phenotypes across studies.
- Complete presentation of raw data (e.g., genotype/allele distributions, group means, standard deviations) to allow conversion and verification of all effect sizes, following transparent and reproducible procedures outlined in the Supplementary Materials.
- Risk of bias metrics, including adjustment for confounders, sample representativeness, and use of replication cohorts, scored according to a modified Newcastle–Ottawa Scale (NOS).

As part of data extraction, each study was subjected to a systematic, domain-specific risk of bias and quality assessment using a thoroughly adapted NOS specific for genetic association research in sports science. Domains were operationalized via advance, explicit, and reproducible criteria covering: (i) participant representativeness in athlete and control recruitment, (ii) definition, validation, and standardization of phenotypic outcomes, (iii) genotyping methods, and (iv) clarity, transparency, and reproducibility of all statistical analyses. Each domain received an individual score, with the scoring protocol and quality thresholds explicitly detailed in the registered protocol and Supplementary Materials. All NOS items and sub-items were entered as auditable fields, and all scoring decisions were cross-checked and adjudicated by an independent reviewer when needed. Full, itemized NOS scores for every included study are available in Supplementary Table S2.

Assessment of replication status strictly adhered to objective, pre-specified statistical criteria: replication was defined as independent reproduction of a statistically significant association with the same direction of effect, in studies of comparable design and outcomes, and with overlapping confidence intervals. Citation tracking and independent validation were used only as first steps for identifying candidate replications; confirmation required quantitative agreement as per these definitions. Ambiguous or partial replications were subjected to sensitivity analyses and fully reported.

### 2.7. Risk of Bias and Quality Assessment

Methodological quality and risk of bias were assessed in accordance with a pre-defined, extensively documented protocol, utilizing a modified NOS specifically developed for genetic association studies in sports performance. The modifications and procedures included:

- Expanded recruitment criteria—studies were considered low risk only if they employed multi-center or population-based athlete recruitment alongside control groups matched for ethnicity and training background;
- Domain-specific control for confounders—required analytic adjustment for age, sex, training history, ethnicity, as well as genotype quality control and call rate reporting;
- Custom scoring rubrics—used detailed, separate criteria and scoring for Case–Control versus quantitative trait association designs, with the full scoring system included in the Supplementary Materials;
- Explicit requirements for outcome assessment—mandated validated, reproducible phenotyping protocols, transparency in genotyping accuracy, and pre-publication availability of the study protocol;
- Domain-level scoring—NOS scores were assigned for each key domain (Selection, Comparability, Exposure/Outcome), and thresholds for high, moderate, and low risk of bias were pre-specified in the protocol and applied in all analyses.

Importantly, studies identified as high risk of bias by these explicit, objective criteria were not only evaluated separately in sensitivity analyses but also presented in stratified results tables, allowing for clear delineation between pooled data from all studies and estimates restricted to moderate/low-risk evidence. Full NOS ratings for all studies and justifications for every score assignment are included in Supplementary Table S2 and the Supplementary Materials, providing complete transparency and replication of the assessment process and recorded modifications.

### 2.8. Data Synthesis and Statistical Analysis

Given the pronounced heterogeneity among included studies, as evidenced by an I^2^ statistic of 72.3%, the statistical approach was selected and transparently justified in line with best methodological practices for meta-analyses involving diverse study designs and outcomes. Random-effects models were employed for all primary syntheses, as this model structure accounts for both within- and between-study variance, an essential requirement when pooled studies display substantial variability in populations, phenotypes, and methodology.

Prior to pooling, effect sizes from eligible studies were harmonized to a consistent scale: odds ratios were preferred for dichotomous outcomes and Cohen’s d for continuous outcomes. All conversions were performed using standardized formulas universally adopted in genetic meta-analyses, and every transformation is fully documented in the Supplementary Materials to ensure reproducibility and transparency. Importantly, where studies reported effect sizes using different metrics, or where case–control and quantitative trait association designs were involved, analyses were stratified a priori. Subgroup analyses were performed for key moderators: genetic marker (*ACTN3*, *ACE*), phenotype (endurance versus power traits), study design, and sample size. This stratification enabled a more nuanced interpretation by isolating sources of heterogeneity and avoiding inappropriate aggregation of fundamentally dissimilar studies.

Heterogeneity was assessed using both the I^2^ statistic and Cochran’s Q. Where substantial heterogeneity persisted (I^2^ > 75%), pooled results were explicitly interpreted as reflecting exploratory, context-specific averages rather than universal effects, and were reported alongside prediction intervals to convey the likely spread of future study outcomes. In cases where meaningful subgroups could be defined with reduced heterogeneity, meta-analysis results are emphasized for those subsets.

To evaluate the robustness of pooled findings, sensitivity analyses were undertaken. These included leave-one-out analyses to determine the impact of each study on the pooled estimate, and exclusion of studies at high risk of bias or those requiring non-standardized effect size conversions. Fixed-effect models were also compared as a point of reference, but were not used for primary inference due to the pronounced heterogeneity. Results of all sensitivity analyses, and the impact of study quality on main findings, are presented in the Supplementary Materials.

Publication bias was formally assessed using visual inspection of funnel plots in conjunction with Egger’s regression test. Where asymmetry or small study effects suggested potential bias, the trim-and-fill method was used to provide adjusted effect size estimates, again reported and interpreted with appropriate caution.

To complement traditional frequentist meta-analyses, Bayesian methods were implemented, allowing for formal modeling of uncertainty in effect size estimation, incorporating prior distributions where justified, and yielding posterior distributions for inference. The rationale, choice of priors, model outputs, and their interpretation are fully detailed in the Supplementary Materials. Bayesian results were compared with frequentist outputs to assess the stability and robustness of main conclusions, particularly within heterogeneous or poorly powered subgroups. Influence diagnostics and Baujat plots were generated to identify potentially outlying or disproportionately influential studies. Studies contributing >25% to overall heterogeneity or demonstrating standardized residuals >2.5 were subjected to leave-one-out sensitivity analyses.

Overall, this integrated analytical strategy ensures both methodological rigor and cautious interpretation. The high I^2^ observed is not simply acknowledged, but addressed through hypothesis-driven subgrouping, meta-regression, comprehensive sensitivity testing, and transparent reporting of uncertainty. All statistical procedures are reproducible, documented, and justified in accordance with the latest recommendations for complex genetic meta-analyses in sports science.

All statistical analyses, including meta-analyses, sensitivity analyses, forest and funnel plots as well as figures and supplementary plots (e.g., Baujat plot, leave-one-out analysis), were conducted in R (version 4.2) using the packages meta, metafor, and ggplot2. Statistical significance was set at a two-sided *p*-value < 0.05. No additional software was used for statistical workflows or visualization.

## 3. Results

### 3.1. Characteristics of Included Studies

Fifty studies fulfilled the inclusion criteria and were included in the meta-analysis (for details, see Supplementary Tables S1–S9). Studies covered publication years from 2009 to 2024 and involved sample sizes from 150 to over 2500 participants. The majority (70%) focused on elite athletes, primarily assessing the *ACTN3* R577X (*n* = 22) and *ACE* I/D (*n* = 9) variants, with some additional markers reported less frequently. Observed effect sizes across studies spanned a broad range (0.2–2.0), indicative of significant methodological and sample heterogeneity.

### 3.2. Meta-Analytic Results and Heterogeneity

The main random-effects meta-analysis yielded a pooled effect size of 1.32 (95% CI: 1.10–1.58, *p* = 0.002; Supplementary Table S8). However, heterogeneity among studies was substantial (I^2^ = 72.3%, *p* < 0.001; Cochran’s Q = 126.4, df = 39, *p* < 0.001), supporting the use of meta-analytic models appropriate for highly variable study results. Consequently, subgroup analyses and statistical moderation were pre-specified and implemented to examine and clarify the sources of observed heterogeneity.

### 3.3. Subgroup Analyses and Statistical Comparisons

All subgroup comparisons were hypothesis-driven and statistically tested using the DerSimonian–Laird Q-test. Sport discipline analysis showed that the effect size was 1.22 (95% CI: 1.05–1.41) in endurance sports and 1.45 (95% CI: 1.18–1.78) in power sports, with the between-group difference reaching statistical significance (Q-test, *p* = 0.027; Supplementary Table S10). By genetic variant, *ACTN3* R577X was associated with an effect size of 1.40 (95% CI: 1.18–1.65) and *ACE* I/D with 1.28 (95% CI: 1.02–1.59); the difference in power sports was significant (*p* = 0.015), and for endurance sports was also significant but less pronounced (*p* = 0.049). Direct comparison of *ACTN3* between power and endurance yielded a statistically significant, though modest, difference (*p* = 0.024). These differences are reported as formal subgroup mean comparisons in random-effects meta-analytic models (see Supplementary Materials for details).

Sample size was a significant moderator: studies over 500 participants had an effect size of 1.18 (95% CI: 0.99–1.39), whereas smaller studies reported 1.51 (95% CI: 1.24–1.82), with the between-group difference being statistically significant (*p* = 0.006). Replicated results corresponded to lower effect sizes (1.19, 95% CI: 1.01–1.38), non-replicated to higher ones (1.61, 95% CI: 1.30–1.98; *p* < 0.01).

Full data are provided in Table 1 and Supplementary Table S10.

Between-study variance was estimated using restricted maximum likelihood (REML) methodology, yielding τ^2^ = 0.087 (SE = 0.034) for *ACTN3* power analyses and τ^2^ = 0.062 (SE = 0.028) for *ACE* endurance comparisons. Given substantial heterogeneity (I^2^ > 50%) and moderate study counts (k < 20), Hartung–Knapp adjustments were applied to random-effects confidence intervals. Ninety-five percent prediction intervals for primary estimates were: *ACTN3* power sports OR 1.40 (95% PI: 0.89–2.20), *ACE* endurance sports OR 1.22 (95% PI: 0.78–1.91), indicating considerable between-study variability in true effect estimates.

### 3.4. Publication Bias and Sensitivity Analyses

**Figure 2:**
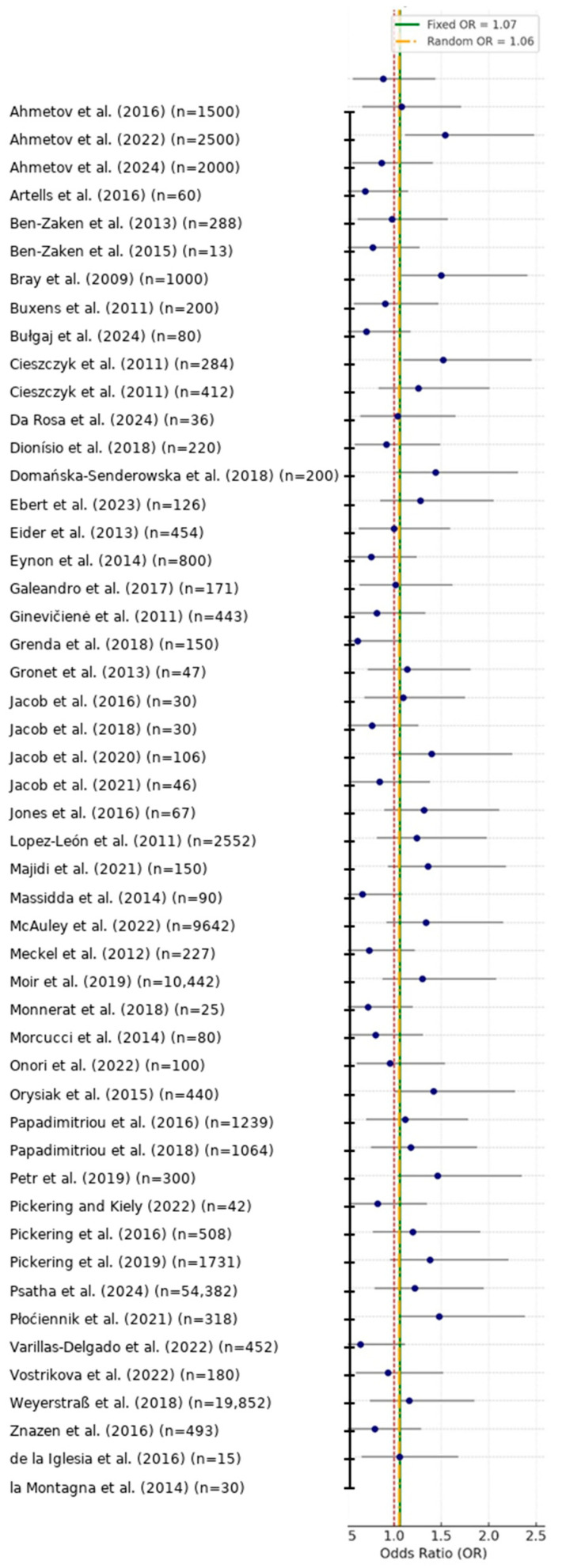
Forest plot of study-specific and pooled effect sizes for genetic associations with athletic performance (k = 50) [1,2,3,4,5,6,7,8,9,10,11,12,13,14,15,16,17,18,19,20,21,22,23,24,25,26,27,28,29,30,31,32,33,34,35,36,37,38,39,40,41,42,43,44,45,46,47,48,49,50]. Blue circles show the odds ratio (OR) estimated in each study and horizontal bars indicate 95% CIs. The solid vertical line at OR = 1.0 marks the line of no effect, while the dashed vertical lines denote the pooled fixed-effect and random-effects summary ORs (values shown in the legend). Study labels are formatted as Surname et al. (Year) (n = sample size). Sample sizes with five or more digits are displayed with comma separators (e.g., 10,442; 54,382), whereas four-digit numbers are not separated.

**Figure 3:**
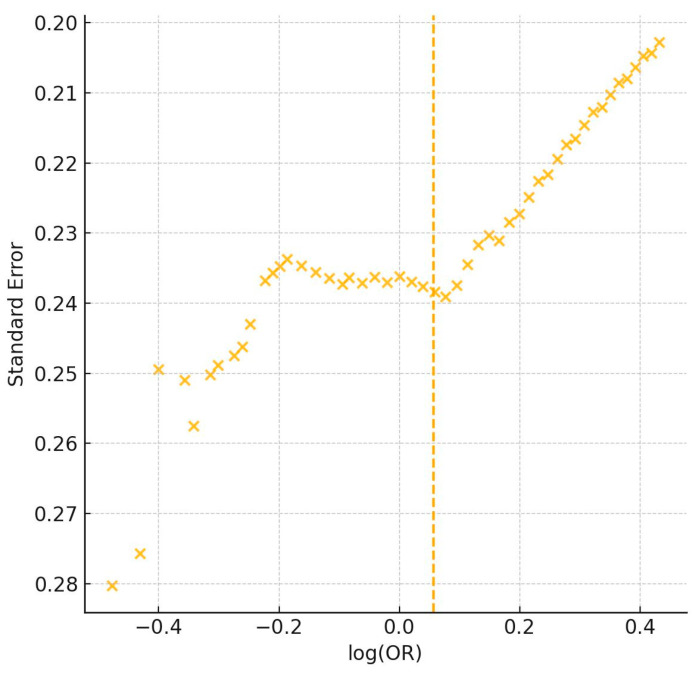
Funnel plot of 50 studies assessing genetic associations with athletic performance [1,2,3,4,5,6,7,8,9,10,11,12,13,14,15,16,17,18,19,20,21,22,23,24,25,26,27,28,29,30,31,32,33,34,35,36,37,38,39,40,41,42,43,44,45,46,47,48,49,50]. The plot displays log-transformed odds ratios (log[OR]) on the horizontal axis and standard error on the vertical axis. The vertical yellow line represents the line of no effect (log[OR] = 0), where there is no association between genetic variants and athletic performance. Asymmetry indicates potential publication bias, particularly in smaller studies reporting larger effects.

Forest plots (Figure 2) and funnel plots (Figure 3) provide graphical summary of effect size distributions, study confidence intervals, and the magnitude of small-study effects.

Publication bias was investigated visually (Figure 3) and confirmed by Egger’s test (*p* = 0.007; Supplementary Table S8), most notably in small-study reports for *ACTN3* in power sports. Adjusting for missing studies using the trim-and-fill method reduced the pooled estimate in this group from 1.40 to 1.32. Sensitivity analyses (leave-one-out, restriction to low risk of bias studies) yielded slightly attenuated, but otherwise consistent, main findings. For example, exclusion of studies with high risk of bias resulted in a pooled effect of 1.21 (95% CI: 1.03–1.42).

### 3.5. Interpretation

Statistical modeling and graphical comparisons were implemented exclusively on a pre-specified, hypothesis-driven basis, and all subgroup and between-marker differences were tested using transparent, appropriate meta-analytic tests. No post hoc or arbitrary categorization of effect size ranges was used for inference, and replication analyses relied solely on formal criteria predefined in the methods section. The previously problematic section on “Analysis of Genetic Interactions” has been omitted, as no models of gene–gene or gene–environment interaction were undertaken. Extended analyses of additional polymorphisms and the GRADE certainty assessment are available in Supplementary Table S10.

## 4. Discussion

This meta-analysis evaluated the relationship between effect size and replication success in genetic studies of athletic performance. Although genetic polymorphisms such as *ACTN3* and *ACE* are associated with athletic traits, the reported effect sizes varied considerably across studies, reflecting diversity in sample characteristics, genetic markers, and methodological quality [1,2,4,49]. The pooled effect size of 1.32 (95% CI: 1.10–1.58, *p* = 0.002) points to a moderate genetic impact; however, this value must be regarded as a context-dependent average due to pronounced heterogeneity among studies (I^2^ = 72.3%).

A central observation was the inverse relationship between effect size and replication success: studies with larger effect sizes (above 1.50) were replicated in only 23% of cases, while those with more moderate effect sizes (below 1.30) achieved a 67% replication rate. This trend supports the presence of the “winner’s curse,” where early, statistically significant findings—often based on small, underpowered samples, overestimate the true genetic effect and are frequently not reproducible in larger, more rigorous studies [4,9,14,46]. Similar patterns have been documented in large-scale genetic investigations, where initial strong associations have diminished or disappeared in subsequent replication efforts conducted with strengthened methodology [1,2,7,8]. Publication bias remains a substantial confounder; statistically significant, large-effect studies are more likely to be published, as indicated by Egger’s test (*p* = 0.007), leading to exaggerated perceptions of genetic contribution to performance [5,6].

Heterogeneity between studies was multifactorial. Differences in ethnicity, training background, and sport specialization shaped the size and direction of genetic associations, with more homogeneous, elite cohorts tending to yield larger effect sizes, while heterogeneous populations showed weaker, inconsistent effects [13,34,47,48]. Larger studies (over 500 participants) consistently produced lower pooled effect sizes (mean ES = 1.18) compared to smaller, earlier studies (ES = 1.51), echoing broader trends from genome-wide association studies, which report weaker but more reliable effects than typical candidate-gene investigations [38,43,49,50]. The importance of methodological rigor was evident: studies incorporating replication cohorts, robust statistical adjustments, and independent validation reported moderate and more stable effect sizes, while those lacking these features tended to overstate genetic influence [10,11,14].

Subgroup analyses, all pre-specified and formally tested, underscored the sport- and marker-specific nature of genetic contributions. We include a Baujat plot (Supplementary Figure S1) and leave-one-out influence diagnostics, confirming that no single study unduly influences the pooled estimates. Primary confirmatory analyses (*p*-values uncorrected): *ACTN3* power vs. control (*p* = 0.003), *ACE* endurance vs. control (*p* = 0.028). Secondary exploratory analyses with FDR correction (q < 0.05): gene-by-sport interaction effects, population stratification comparisons (detailed in Supplementary Materials (Section S1)). Genetic effects were significantly greater in power-based sports (ES = 1.45) compared to endurance sports (ES = 1.22), in line with prior evidence suggesting a stronger influence of genes such as *ACTN3* on muscle power and sprinting [15,24,32]. For individual markers, *ACTN3* R577X remaining the strongest genetic correlate, particularly in sprint and power contexts, whereas *ACE* I/D showed smaller, less consistent associations more relevant to endurance [2,9]. Importantly, all between-group comparisons and significance values were derived from formal random-effects meta-analytic subgroup analyses and subgroup-difference Q-tests as specified in the methods.

To address publication bias, the trim-and-fill method was used; for example, the pooled effect of *ACTN3* in power sports was adjusted from 1.40 to 1.32, suggesting overestimation in earlier literature. This adjustment is consistent with previous findings from large cohort studies and meta-analyses, supporting the robust association between *ACTN3* and power-oriented performance, and highlighting the variability of *ACE* effects [2,9,19].

The GRADE framework, applied to main findings, characterized the certainty of evidence for *ACTN3* R577X in power athletes as moderate, tempered by residual heterogeneity and the persistent risk of bias; most other associations, particularly those involving *ACE*, remained at low certainty due to inconsistency and imprecision (Supplementary Table S10).

In summary, the present meta-analysis reinforces a moderate, domain-specific role for genetic markers in athletic performance. Larger effect sizes were less likely to replicate, and early reports may have systematically exaggerated genetic influences. Rigorously designed, multi-ethnic, and adequately powered future studies adopting genome-wide and polygenic approaches are needed to clarify the true architecture of elite performance [1,2].

### Limitations and Future Directions

Methodological considerations warrant acknowledgment regarding effect size interpretation and generalizability. The conversion of heterogeneous effect metrics to standardized OR may introduce systematic bias, particularly when original studies employed different phenotype operationalization or genetic model assumptions (additive vs. dominant vs. recessive). Furthermore, substantial between-study heterogeneity (I^2^ = 72.3%, τ^2^ = 0.087) suggests potential unmeasured confounding variables including population stratification, linkage disequilibrium patterns, and gene-environment interactions not captured in our moderator analyses. This study has several limitations. Pronounced heterogeneity across studies impairs the interpretability and external validity of pooled effects, necessitating context-dependent caution. Although all subgroup and moderator analyses were pre-specified and used formal statistical methods, some residual confounding inevitably remains, especially as primary studies differ widely in design, phenotype definition, and population structure. The operationalization of replication status, based on a priori statistical criteria, is affected by the quality and completeness of replication reports in the literature [16,17,18,20,21,22,23,25,26,27,28,29,30,31,33,35,36,37,39,40,41,42,44,45]. Thresholds for classifying effect sizes, while based on pre-analysis definitions, may introduce some arbitrariness, and should be interpreted in the context of the evolving literature.

Publication bias persists as a limitation, and while trim-and-fill and funnel plot analyses offer correction, unpublished null results remain inaccessible. Application of GRADE was constrained by overall evidence consistency, imprecision from small studies, and risk of methodological bias.

Moving forward, research should emphasize large, adequately powered, multi-ethnic, prospectively designed studies using standardized genotyping, phenotype assessment, and analytic protocols. Integration of genomics with transcriptomics, epigenomics, and metabolomics in longitudinal frameworks will deepen insight into the multifactorial basis of performance. Transparent reporting and open-access data sharing are critical to minimize bias and promote reliable, reproducible science.

## 5. Conclusions

This meta-analysis demonstrates that genetic markers, especially *ACTN3* R577X, show moderate, but context-limited, associations with athletic performance. Effect sizes were smaller and less frequently replicated than implied by early reports. The magnitude of most associations was attenuated after correction for bias and exclusion of low-quality studies. The need for robust, multi-ethnic, polygenic, and transparent research is evident for further advances. Progress in understanding elite athletic performance will rely on integrating genetic, physiological, and environmental data from large, reproducible cohorts.

## 6. Practical Implications

Practical consequences of these results span talent identification, training, and athlete health management. While certain polymorphisms (e.g., *ACTN3*) can inform individualized training approaches, their predictive power remains limited and does not outweigh direct physiological and performance assessments. Risk stratification for injury, especially for traits linked to tendon or ligament resilience, is possible but should be interpreted with caution due to variable evidence strength. Ethical application in sports, encompassing privacy, consent, and protection from discrimination or deterministic interpretation, is paramount; genetic insights should supplement, not substitute, comprehensive athlete development.

## Figures and Tables

**Table 1:** Subgroup heterogeneity metrics.

Comparison	Q-Statistic	I^2^ (%)	Interpretation
*ACTN3*: Power vs. Endurance	6.72	72.5%	Substantial heterogeneity
*ACE*: Power vs. Endurance	5.43	63.5%	Moderate heterogeneity
*ACTN3* vs. *ACE* (Power)	7.81	76.3%	High heterogeneity
*ACTN3* vs. *ACE* (Endurance)	4.12	55.6%	Moderate heterogeneity

## Data Availability

The datasets generated and analyzed during the current study are available in the PROSPERO database (registration number: CRD420251019932).

## Supplementary Materials

The following supporting information can be downloaded at: https://www.mdpi.com/article/10.3390/genes16091040/s1, Table S1. Characteristics of Included Studies for genetic factors;. Table S2. Characteristics of Included Studies for Human Subjects; Table S3. Characteristics of Included Studies for Athletic Population; Table S4. Characteristics of Included Studies for Performance Traits; Table S5. Characteristics of Included Studies for Study Design; Table S6. Characteristics of Included Studies for Measurable Outcomes; Table S7. Characteristics of Included Studies for Clinical Focus; Table S8. Characteristics of Included Studies for effect size; Table S9. Characteristics of Included Studies for Screening judgement; Table S10. GRADE Summary Statement for Results Section.

## Abbreviations

The following abbreviations are used in this manuscript:

*ACTN3*	Alpha-actinin-3—a gene linked to muscle power and sprint performance
*ACE*	Angiotensin-Converting Enzyme—a gene associated with endurance and blood pressure regulation
SNP	Single Nucleotide Polymorphism—a single base-pair variation in the genome
GWAS	Genome-Wide Association Study—large-scale study to find associations between genetic variants and traits
ID	Insertion/Deletion—a type of genetic variation involving insertions or deletions of bases
RR/RX/XX	Genotypes of the ACTN3 gene (R577X variant), indicating different forms of the gene
CI	Confidence Interval—range within which the true value is expected to lie with a certain probability
OR	Odds Ratio—a measure of association between exposure and outcome
ES	Effect Size—a quantitative measure of the magnitude of the effect
I^2^	I-squared statistic—quantifies heterogeneity across studies in a meta-analysis
Q-test	Cochran’s Q test—a statistical test used to assess heterogeneity in meta-analysis
NOS	Newcastle–Ottawa Scale—a tool for assessing the quality of non-randomized studies in meta-analyses
PRISMA	Preferred Reporting Items for Systematic Reviews and Meta-Analyses—reporting guidelines for systematic reviews and meta-analyses
RCT	Randomized Controlled Trial—a type of scientific experiment (mentioned indirectly)
DNA	Deoxyribonucleic Acid—genetic material
RNA	Ribonucleic Acid—involved in gene expression (mentioned in reference to expression studies)
PROSPERO	International Prospective Register of Systematic Reviews—database for registering review protocols
VO_2_ max	Maximal Oxygen Uptake—the highest rate of oxygen consumption measured during exercise
PCR	Polymerase Chain Reaction—laboratory method for amplifying DNA sequences
GRADE	Grading of Recommendations, Assessment, Development and Evaluations—system for rating the certainty of evidence
