# Supplementary material for: Effect Size and Replicability in Genetic Studies of Athletic Performance: A Meta-Analytical Review
Source: Genes (Basel). 2025 Aug 31;16(9):1040. doi: 10.3390/genes16091040 (PMC12469432; doi:10.3390/genes16091040)
Supplement: Supplementary file 1 [file genes-16-01040-s001.zip › genes-3817896-supplementary.pdf]

Supplementary materials

Section S1

Table S1. Characteristics of Included Studies for genetic factors

Author(s) & Year	Genetic Factors
Ahmetov et al., 2022	The paper extensively examines genetic factors, including specific SNPs and genetic variations (220 DNA polymorphisms) directly related to athletic performance.
Massidda et al., 2014	The study examines specific genetic polymorphisms (ACE, ACTN-3, BDKRB2, VDR-ApaI, VDR-BsmI, and VDR-FokI) and their direct relation to athletic performance.
Dionísio et al., 2017	The study examines multiple specific genetic polymorphisms (ACTN3, AMPD1, ACE, AGT) and establishes a direct relation to athletic performance.
De la Iglesia et al., 2020	The study explicitly examines genetic factors, specifically developing a "genetic endurance prediction score (GES)" and mentions single nucleotide polymorphisms.
Ahmetov et al., 2016	The paper is centrally focused on genetic factors, specifically DNA polymorphisms and their relation to athletic performance, with detailed discussion of genetic markers.
La Montagna et al., 2019	The study explicitly examines genetic polymorphisms in five specific genes (ACTN3, COL5A1, MCT1, VEGF, and HFE) in relation to athletic performance.
Majidi et al., 2021	The study specifically examines the Alpha-actin3 gene, its variants (RR, RX, XX genotypes), and SNPs in relation to athletic performance. It directly links genetic variations to performance outcomes.
Buxens et al., 2009	The study explicitly examines 36 genetic variants within 20 different genes, including specific polymorphisms (rs1800795, rs1208, rs2070744) directly related to athletic performance.
Vostrikova et al., 2022	The study explicitly examines specific genetic factors (SNPs in ACE, BDKRB2, PPARGC1A, and NOS3 genes) in direct relation to athletic performance.
McAuley et al., 2022	The study explicitly examines genetic factors, including 99 polymorphisms within 63 genes, and their direct relation to athletic performance (injury risk).
Psatha et al., 2024	The study explicitly examines genetic variants (including ACE and ACTN3 genes) in relation to athletic performance.
Ginevičienė et al., 2021	The study directly examines specific genetic polymorphisms (ACE and ACTN3) and their connection to athletic performance, including genetic analysis methodology.
Monnerat et al., 2018	The study examines 10 genetic polymorphisms related to performance, including SNP analysis and population genetics data, directly relating to athletic performance.
Varillas-Delgado et al., 2022	The study extensively examines multiple genetic factors (polymorphisms) directly related to athletic performance, including liver metabolizers, iron metabolism and energy efficiency genes, cardiorespiratory fitness genes, and muscle injury-related genes.
Ben-Zaken et al., 2013	The study examines multiple specific genetic polymorphisms (ACTN3C/T, ACEI/D, etc.) in direct relation to athletic performance.
Jacob et al., 2016	The study examines specific genetic polymorphisms (ACE, BDNF, DRD2, COMT) and establishes a direct relationship between these genetic variations and athletic performance.
Onori et al., 2022	The study explicitly examines multiple genetic factors (ACE, ACTN3, COL1A1, and MCT1) in relation to athletic performance and injury risk.
Ben-Zaken et al., 2017	The study examines multiple genetic polymorphisms (9 endurance-related, 5 power-related) and establishes a direct relation between these genetic factors and athletic performance.
Jacob et al., 2021	The study examines specific genetic variants (ACE, ACTN3, ADRB1, PPARGC1A) and their direct relation to athletic performance, with the SNP identification (rs numbers) provided.
Jacob et al., 2019	The study directly examines specific genetic polymorphisms (ACTN3 R577X and ACE I/D) in relation to athletic performance at the elite level, meeting the genetic factors criterion.

Jacob et al., 2018	Multiple specific genetic polymorphisms (ADRB2, PPARGC1A, ACE, COMT, BDNF, ADRB1, ADRB3, DRD2) are examined, and their direct relation to performance is established.
Galeandro et al., 2020	The study directly examines specific genetic factors (ACTN3 and ACE polymorphisms) in relation to athletic performance, plus mitochondrial DNA content.
Da Rosa et al., 2022	The study specifically examines ACE and ACTN3 gene variants, with DNA analysis from peripheral blood, and directly relates these genetic factors to athletic performance.
Pickering & Kiely, 2019	The study directly examines genetic factors (multiple genetic variants) in relation to athletic performance, specifically analyzing both endurance and speed-power related variants.
Artells et al., 2016	The study specifically examines single nucleotide polymorphisms in the Elastin gene (ELN) and their relationship to athletic performance/injury.
Ebert et al., 2023	The study examines specific SNPs and their direct relation to athletic performance, indicating it investigates genetic factors in relation to athletic performance.
Lopez-León et al., 2017	The study specifically examines the PPARA gene intron 7 G/C polymorphism (rs4253778) in relation to athletic performance.
Pickering et al., 2021	The study examines specific SNPs, ranging from 600K to 1.14M, and directly relates these genetic factors to athletic performance.
Gronek et al., 2020	The study specifically examines the ACE I/D genetic polymorphism, using direct genetic analysis through polymerase chain reaction, and clearly connects the genetic factors to performance outcomes.
Znazen et al., 2019	The study directly examines genetic factors, specifically the ACE gene ID polymorphism, in relation to athletic performance.
Ahmetov et al., 2024	The paper is centrally focused on genetic factors, specifically DNA polymorphisms and their relationship to athletic performance and physical activity.
Moir et al., 2020	The study explicitly examines genetic factors, identifying 160 polymorphisms in 27 genes, including 16 SNPs in 14 genes associated with marathon running performance.
Petr et al., 2020	The study explicitly examines specific genetic factors (PPARs and their variants) in direct relation to athletic performance, including specific SNPs (rs1801282, rs4253778, rs8192678, rs2016520).
Bray et al., 2008	The paper extensively covers genetic factors, including "214 autosomal gene entries and quantitative trait loci" plus X chromosome and mitochondrial genes, all directly related to performance.
Weyerstraß et al., 2017	The study examines specific genetic polymorphisms (ACE, ACTN3, AGT, etc.) in direct relation to athletic performance.
Bulğay et al., 2024	The study specifically examines the rs17602729 polymorphism in the AMPD1 gene using whole exome sequencing.
Morucci et al., 2015	The study examines specific genetic polymorphisms (ACE, ACTN3, VDR) and establishes a direct relation to athletic performance. The genetic testing methodology is described.
Jones et al., 2018	The study examines 15 performance-associated gene polymorphisms and directly relates them to athletic performance, meeting the genetic factors criterion.
Pickering et al., 2018	The study examines specific SNPs (VEGF rs2010963, ADRB2 rs1042713, etc.) and their direct relation to athletic performance, using a genetic algorithm for prediction.
Meckel et al., 2020	The study specifically examines the ACSL A/G polymorphism and its direct relation to athletic performance.
Papadimitriou et al., 2016	The study specifically examines the ACTN3 R577X and ACE I/D genetic variants and their direct relationship to sprint performance, indicating that it examines genetic factors in relation to athletic performance.
Eynon et al., 2014	The study directly examines a specific genetic polymorphism (ACTN3 R577X) in relation to athletic performance.
Cieszczyk et al., 2011	The study explicitly examines the R577X polymorphism of the ACTN3 gene and its relationship to athletic performance.

Cięszczyk et al., 2011	The study explicitly examines a specific genetic factor (ACTN3 R577X polymorphism) in relation to athletic performance, meeting the genetic factors criterion.
Płóciennik et al., 2018	The study examines specific genetic polymorphisms (seven PEPs) in direct relation to athletic performance, including detailed analysis of interactions between genes (ACTN3 and SNAP-25).
Grenda et al., 2014	The study explicitly examines specific genetic factors (ACE ID and ACTN3 R577X polymorphisms) in direct relation to athletic performance.
Papadimitriou et al., 2018	The study examines specific genetic variants (ACTN3 R577X and ACE I/D) and their direct relation to athletic performance, indicating that it meets the genetic factors criterion.
Orysiak et al., 2015	The study directly examines the ACTN3 R577X polymorphism in relation to athletic performance.
Domańska-Senderowska et al., 2019	The study examines ACTN3 gene expression in relation to athletic performance.
Eider et al., 2013	The study directly examines genetic factors, specifically the ACE gene insertion/deletion polymorphism (I/D) and its relationship to athletic performance.

Table S2. Characteristics of Included Studies for Human Subjects

Author(s) & Year	Human Subjects
Ahmetov et al., 2022	The study focuses on "elite athletes" and athlete status, clearly indicating human subjects.
Massidda et al., 2014	The study explicitly uses human subjects, specifically male Italian soccer players (n=90), and does not involve animal models.
Dionísio et al., 2017	The study explicitly states that it uses 220 young male athletes as participants, indicating it involves human subjects rather than animal models.
De la Iglesia et al., 2020	The study explicitly mentions "fifteen male cyclists" as subjects, confirming that it involves human participants.
Ahmetov et al., 2016	The study focuses on human athletes, with specific mention of different human populations (African-American, Jamaican, Japanese, and Russian athletes).
La Montagna et al., 2019	The study clearly involves human subjects - 30 professional soccer players.
Majidi et al., 2021	The study clearly involves human subjects, including athletes and a control group. No animal models are mentioned.
Buxens et al., 2009	The study explicitly involves human subjects - world-class endurance (N=100) and power male athletes.
Vostrikova et al., 2022	The study clearly involves human subjects (martial arts athletes), with no animal models mentioned.
McAuley et al., 2022	The study explicitly involves human subjects (football players), with 9,642 participants across all analyzed studies.
Psatha et al., 2024	The study clearly involves human subjects, with explicit mention of 54,382 subjects including 11,501 athletes and 42,881 controls.
Ginevičienė et al., 2021	The study explicitly involves 193 Lithuanian elite athletes and 250 human controls, indicating that it involves human subjects rather than animal models.
Monnerat et al., 2018	The study explicitly uses 25 male professional soccer players as human subjects, with no animal models involved.
Varillas-Delgado et al., 2022	The study involves 452 human subjects, including 292 professional athletes and 160 non-athletes.

Ben-Zaken et al., 2013	The study explicitly involves human subjects, including 82 power-speed athletes, 87 endurance athletes, and 119 non-athletic controls.
Jacob et al., 2016	The study explicitly states that it uses 30 sub-elite Australian Football players as human subjects, with no mention of animal models.
Onori et al., 2022	The study explicitly involves 100 male professional and semi-professional rugby players, plus a control group of non-athletic males.
Ben-Zaken et al., 2017	The study explicitly involves 13 elite middle-distance runners, indicating that it involves human subjects rather than animal models.
Jacob et al., 2021	The study involves human subjects, specifically 46 AFL (Australian Football League) players, with no animal models involved.
Jacob et al., 2019	The study clearly involves human subjects, specifically 47 AFL players and 59 healthy controls.
Jacob et al., 2018	The study explicitly involves 30 Australian Rules Football players, with no mention of animal models.
Galeandro et al., 2020	The study explicitly involves human subjects: soccer players (n=43) and healthy non-athletic controls (n=128).
Da Rosa et al., 2022	The study explicitly involves 36 human participants, with clear demographic information provided (15 girls, 21 boys, age 16.4 ± 1.2 years).
Pickering & Kiely, 2019	The study explicitly involves human subjects - 5 elite track-and-field athletes and 503 White European controls.
Artells et al., 2016	The study involves 60 top-class football players, which meets the requirement for human subjects.
Ebert et al., 2023	The study explicitly involves 126 Estonian National Team members, who are Olympic athletes and International Championships participants. There is no mention of animal models, indicating the study involves human subjects.
Lopez-León et al., 2017	The study explicitly involves human subjects, comparing endurance athletes (n=760) to controls (n=1792).
Pickering et al., 2021	The study involves human subjects, including 1,206 elite youth football players as well as additional cohorts of 126 Polish women and 399 Russian athletes.
Gronek et al., 2020	The study explicitly involves 47 elite male field hockey players, indicating that it involves human subjects and not animal models.
Znazen et al., 2019	The study explicitly involves human subjects - 282 Tunisian athletes and 211 sedentary volunteers as controls.
Ahmetov et al., 2024	The study focuses on human athletes and physical activity in humans, with no mention of animal models.
Moir et al., 2020	The study involves human subjects, specifically 10,442 participants including 2,984 marathon runners, and does not mention any animal models.
Petr et al., 2020	The study focuses on elite athletes and human performance, without mentioning any animal models.
Bray et al., 2008	The study involves human subjects, as the abstract explicitly refers to the "human gene map" and human variation in fitness traits, without mentioning any animal models.
Weyerstraß et al., 2017	The study involves human subjects, with 5,834 power athletes and 14,018 controls mentioned explicitly.
Bulğay et al., 2024	The study explicitly involves human subjects, including 60 elite athletes and 20 control individuals.
Morucci et al., 2015	The study explicitly involves 80 Italian male gymnasts, with no animal models used.
Jones et al., 2018	The study explicitly uses male athletes as participants, with two separate cohorts (n=28 and n=39), indicating it involves human subjects and not animal models.

Pickering et al., 2018	The study explicitly uses 42 male soccer players as human subjects, and provides clear demographic information about the participants, including their age, height, and weight.
Meckel et al., 2020	The study explicitly involves 167 male athletes and 60 controls, indicating that it involves human subjects and not animal models.
Papadimitriou et al., 2016	The study explicitly involves 346 elite human sprinters from multiple countries and ethnic backgrounds, indicating that it involves human subjects rather than animal models.
Eynon et al., 2014	The study explicitly involves human subjects - European team-sport athletes, endurance athletes, sprint/power athletes, and controls.
Cieszczyk et al., 2011	The study explicitly involves human subjects: 80 male Polish rowers and 204 control volunteers.
Cięszczyk et al., 2011	The study explicitly involves human subjects, including 158 power-oriented athletes and 254 control volunteers.
Płóciennik et al., 2018	The study clearly involves human subjects: 27 elite gymnasts, 46 sub-elite gymnasts, and 245 sedentary controls.
Grenda et al., 2014	The study explicitly involves human subjects (swimmers and control subjects), with DNA samples taken from oral epithelial cells.
Papadimitriou et al., 2018	The study explicitly involves 698 male and female Caucasian athletes, indicating that it involves human subjects rather than animal models.
Orysiak et al., 2015	The study explicitly involves human subjects: 86 European white male athletes and 354 nonathletic controls.
Domańska-Senderowska et al., 2019	The abstract refers to "athletes," indicating human subjects.
Eider et al., 2013	The study explicitly involves human subjects, including 100 Polish power athletes and 354 sedentary volunteers as controls.

Table S3. Characteristics of Included Studies for Athletic Population

Author(s) & Year	Athletic Population
Ahmetov et al., 2022	The study specifically focuses on elite athletes and athletic performance.
Massidda et al., 2014	The study participants are clearly defined as "top-level male Italian soccer players", an athletic population.
Dionísio et al., 2017	The participants are described as professional minor league soccer team athletes, clearly indicating an athletic population.
De la Iglesia et al., 2020	The study population consists of cyclists, clearly meeting the athletic population criterion.
Ahmetov et al., 2016	The study specifically focuses on elite athletes and athlete status across multiple populations.
La Montagna et al., 2019	The study population consists of professional soccer players, which clearly meets the criterion for athletic population.
Majidi et al., 2021	The study explicitly focuses on athletes, including both sprinters and endurance athletes, with a control group for comparison.
Buxens et al.,	The study population consists of world-class athletes, specifically focusing on endurance

2009	and power athletes at the highest level of performance.
Vostrikova et al., 2022	The study specifically focuses on martial arts athletes, clearly meeting this criterion.
McAuley et al., 2022	The study specifically focuses on football players, which clearly meets the athletic population criterion.
Psatha et al., 2024	The study explicitly includes athletes, specifically mentioning endurance and power athletes (11,501 athletes in the sample).
Ginevičienė et al., 2021	The study focuses specifically on elite athletes and includes an appropriate control group from the general population.
Monnerat et al., 2018	The participants are explicitly described as "professional soccer players" from a Brazilian first-division soccer club, meeting the criteria for an athletic population.
Varillas-Delgado et al., 2022	The study specifically focuses on professional athletes, including elite endurance athletes (cyclists and runners) and professional football players.
Ben-Zaken et al., 2013	The study specifically focuses on power-speed athletes and endurance athletes, with a control group of non-athletes.
Jacob et al., 2016	The participants are clearly described as sub-elite Australian Football players, indicating they are an athletic population.
Onori et al., 2022	The study population consists of professional and semi-professional rugby players, clearly meeting this criterion.
Ben-Zaken et al., 2017	The participants are explicitly described as "elite middle-distance runners" with a clear athletic training background from age 14-15, indicating that the study involves an athletic population.
Jacob et al., 2021	The participants are elite athletes from the Australian Football League, which is a professional team sport.
Jacob et al., 2019	The study specifically focuses on elite athletes from an Australian Football League (AFL) club, meeting the criterion for an athletic population.
Jacob et al., 2018	The participants are sub-elite Australian Rules Football players, clearly an athletic population.
Galeandro et al., 2020	The study clearly focuses on elite soccer players, which meets the criterion for athletic population.
Da Rosa et al., 2022	The participants are explicitly described as school-level competitors, including athletes from specific disciplines (sprint, jump, endurance), and their training experience is reported (4 ± 1.2 years).
Pickering & Kiely, 2019	The study clearly includes elite athletes, including an Olympic champion, meeting this criterion fully.
Artells et al., 2016	The study specifically focuses on "top class football players," which meets the requirement for an athletic population.
Ebert et al., 2023	The study cohort consists of a high-level athletic population, including Olympic athletes and International Championships participants, indicating they are elite athletes or regularly physically active individuals.
Lopez-León et al., 2017	The study specifically focuses on "endurance athletes" as the target population.
Pickering et al., 2021	The study participants are elite athletes, including youth football players and Russian athletes, indicating the study focuses on an athletic population.
Gronek et al., 2020	The study focuses on elite male professional field hockey players, clearly defining the athletic population.
Znazen et al., 2019	The study specifically focuses on athletes, with clear categorization into endurance (N=149) and power (N=133) athletes, including elite subgroups.
Ahmetov et al.,	The study explicitly focuses on athletes and physical activity, including specific athlete

2024	status categories (endurance, power, and strength-related).
Moir et al., 2020	The study specifically focuses on elite marathon athletes and marathon runners, meeting the criterion for an athletic population.
Petr et al., 2020	The study specifically focuses on elite athletes and compares them with sub-elite athletes.
Bray et al., 2008	The study covers both "sedentary or active people" and includes exercise responses and training-induced adaptations, indicating that the study population includes athletes or regularly physically active individuals.
Weyerstraß et al., 2017	The study specifically focuses on power athletes, which directly meets the criterion for an athletic population.
Bulğay et al., 2024	The study specifically focuses on elite athletes, including both sprinters/power athletes and endurance athletes.
Morucci et al., 2015	The study focuses on "high level gymnasts", an elite athletic population that is clearly defined. The participants are competitive athletes.
Jones et al., 2018	The participants are explicitly described as athletes, including athletes from different sports and soccer players, and all subjects are engaged in resistance training programs, indicating the study uses an athletic population.
Pickering et al., 2018	The participants are youth soccer players, an actively training population in a sports-specific context.
Meckel et al., 2020	The study focuses on clear athletic populations, including soccer players, sprinters, jumpers, and long-distance runners.
Papadimitriou et al., 2016	The study focuses exclusively on elite sprinters, including Olympic-level athletes, with a large sample size of 555 performance times from 346 athletes, indicating that the study population is an athletic population.
Eynon et al., 2014	The study specifically focuses on elite team-sport athletes, endurance athletes, and sprint/power athletes.
Cieszczyk et al., 2011	The study specifically focuses on rowers of nationally competitive standard, including both elite and non-elite athletes.
Cięszczyk et al., 2011	The study specifically examines power-oriented athletes, including sprinters, meeting the criterion of an athletic population.
Płóciennik et al., 2018	The study directly studies elite and sub-elite gymnasts, clearly meeting the athletic population criterion.
Grenda et al., 2014	The study specifically focuses on elite swimmers, including both short distance and long distance swimmers, which clearly meets this criterion.
Papadimitriou et al., 2018	The study specifically examines endurance runners, including high-level athletes within 20% of world records, indicating that it focuses on an athletic population.
Orysiak et al., 2015	The study specifically focuses on canoe sprint athletes (canoe and kayak paddlers).
Domańska-Senderowska et al., 2019	The study explicitly mentions "athletes" as participants.
Eider et al., 2013	The study specifically focuses on "elite Polish athletes of the highest nationally competitive standard" and even stratifies them into sub-elite, elite, and top-elite categories.

Table S4. Characteristics of Included Studies for Performance Traits

Author(s) & Year	Performance Traits

Ahmetov et al., 2022	The study clearly investigates genetic influences on specific sports-related traits, including endurance, power, strength, and injury risk.
Massidda et al., 2014	The study investigates vertical jump performance, a specific sports-related trait, and examines gene-performance relationships, including implications for training and injury susceptibility.
Dionísio et al., 2017	The study investigates specific sports-related traits such as jumping, sprinting, and endurance, and mentions gene-environment interactions in athletic performance.
De la Iglesia et al., 2020	The study investigates genetic influences on endurance performance and includes analysis of sport efficiency variables.
Ahmetov et al., 2016	The study examines genetic influences on specific sports-related traits, particularly endurance and power/strength-related characteristics.
La Montagna et al., 2019	The study investigates genetic influences on specific sports-related traits including performance enhancement and injury susceptibility, and considers gene-environment interactions through personalized training and nutrition programs.
Majidi et al., 2021	The study investigates specific sports-related traits including muscle mass, physical fitness, and performance in different types of athletics (sprinting vs. endurance).
Buxens et al., 2009	The study investigates genetic influences on specific sports-related traits, particularly distinguishing between endurance and power performance.
Vostrikova et al., 2022	The study investigates genetic influences on specific sports-related traits including endurance, speed, and power qualities in martial arts athletes.
McAuley et al., 2022	The study investigates genetic influences on specific sports-related traits, particularly focusing on injury risk and susceptibility, which is a crucial performance-related outcome.
Psatha et al., 2024	The study specifically investigates genetic influences on endurance and power performance traits.
Ginevičienė et al., 2021	The study investigates specific sports-related traits (muscle strength, power) and examines the genetic influence on these performance measures, linking genotypes to performance outcomes.
Monnerat et al., 2018	The study investigates multiple sports-related traits, such as hypertrophy, energy expenditure, VO2max, and recovery, examining the genetic influences on these specific performance traits.
Varillas-Delgado et al., 2022	The study investigates genetic influences on specific sports-related traits including endurance performance, cardiorespiratory fitness, energy efficiency, and muscle injury susceptibility.
Ben-Zaken et al., 2013	The study investigates genetic influences on specific sports-related traits, particularly power-speed and endurance capabilities.
Jacob et al., 2016	The study investigates specific sports-related traits, such as endurance, power, and technical skill, and examines the gene-performance relationships for these performance domains.
Onori et al., 2022	The study investigates genetic influences on injury risk, which is a key performance-related trait in sports.
Ben-Zaken et al., 2017	The study investigates the genetic influence on endurance performance and the transition from middle- to long-distance running, focusing on specific sports-related traits.
Jacob et al., 2021	The study investigates endurance performance specifically, with a clear connection between the genetic variants and the performance outcome in the sport-specific context.
Jacob et al., 2019	While specific performance traits aren't directly measured, the study investigates genetic variants known to be associated with athletic performance in elite sport, which is relevant to the performance traits criterion.
Jacob et al., 2018	The study investigates specific sports-related traits, including aerobic performance (time trials), sport-specific skills (handball, kicking), match performance metrics, and motor learning aspects.
Galeandro et al., 2020	The study investigates genetic influences on athletic performance in soccer players and includes analysis of training effects on genetic markers.

Da Rosa et al., 2022	The study investigates specific sports-related traits, including sprint ability, jumping power, and endurance, and examines the relationships between these performance traits and genetic factors.
Pickering & Kiely, 2019	The study investigates genetic influences on specific sports-related traits (endurance and speed-power capabilities).
Artells et al., 2016	The study investigates genetic influences on injury risk and recovery time, which are relevant sports-related traits.
Ebert et al., 2023	The study investigates both performance (medalist status) and injury risk factors, examining gene-performance relationships for specific sports-related traits.
Lopez-León et al., 2017	The study investigates genetic influences on endurance performance, which is a specific sports-related trait.
Pickering et al., 2021	The study specifically investigates sprint performance, including muscle fiber type analysis, and examines the relationship between genetic factors and athletic performance traits.
Gronek et al., 2020	The study investigates multiple specific sports-related traits, including endurance, power, speed, and recovery, and analyzes the gene-performance relationship.
Znazen et al., 2019	The study investigates genetic influences on specific sports-related traits, distinguishing between endurance and power performance.
Ahmetov et al., 2024	The study explicitly investigates genetic influences on sports-related traits, including endurance, power, and strength, as well as responses to training.
Moir et al., 2020	The study specifically investigates genetic influences on endurance-related traits and marathon running performance.
Petr et al., 2020	The study investigates genetic influences on specific sports-related traits including strength, power, endurance, and performance in different sports disciplines.
Bray et al., 2008	The study specifically examines genetic influences on "fitness and performance phenotypes" including responses to acute exercise and training adaptations.
Weyerstraß et al., 2017	The study investigates genetic influences on power athlete status, which is a specific sports-related trait.
Bulğay et al., 2024	The study investigates genetic influences on specific sports-related traits, differentiating between sprint/power and endurance performance.
Morucci et al., 2015	The study investigates genetic influences on specific sports-related traits, including power features and apparatus-specific performance, and analyzes clear gene-performance relationships.
Jones et al., 2018	The study investigates power and endurance qualities, as well as gene-training interactions, meeting the performance traits criterion.
Pickering et al., 2018	The study investigates aerobic fitness adaptations, examining gene-training interaction and sport-specific performance outcomes.
Meckel et al., 2020	The study investigates endurance trainability and links genetic factors to VO2 max response, which are relevant to sports-related traits.
Papadimitriou et al., 2016	The study investigates sprint performance specifically, examining the gene-performance relationships and quantifying the impact on this athletic trait.
Eynon et al., 2014	While specific performance traits aren't extensively detailed, the study examines genetic influences on team-sport athletic status and compares different athletic categories (team-sport, endurance, sprint/power).
Cieszczyk et al., 2011	The study investigates genetic influences on rowing performance, specifically examining power ability and endurance performance.
Cięszczyk et al., 2011	The study investigates genetic influences on specific sports-related traits, namely sprint and power performance, meeting the performance traits criterion.
Płóciennik et al., 2018	The study investigates genetic influences on gymnastic aptitude and muscle architecture, including the interaction between muscle-related genes and nervous system genes.
Grenda et al.,	The study investigates genetic influences on specific sports-related traits, namely sprint and

2014	endurance performance in swimming.
Papadimitriou et al., 2018	The study investigates endurance performance specifically and the connection between genetic variants and running performance, indicating that it meets the performance traits criterion.
Orysiak et al., 2015	The study investigates genetic influences on specific sports-related performance traits (sprint performance at different distances).
Domańska-Senderowska et al., 2019	The study specifically investigates explosive strength through jump tests (SJ and CMJ) and its relationship with genetic factors.
Eider et al., 2013	The study investigates genetic influences on power performance specifically, with clear theoretical mechanisms proposed (D allele associated with higher ACE activity potentially benefiting power performance).

Table S5. Characteristics of Included Studies for Study Design

Author(s) & Year	Measurable Outcomes
Ahmetov et al., 2022	The study clearly investigates genetic influences on specific sports-related traits, including endurance, power, strength, and injury risk.
Massidda et al., 2014	The study investigates vertical jump performance, a specific sports-related trait, and examines gene-performance relationships, including implications for training and injury susceptibility.
Dionísio et al., 2017	The study investigates specific sports-related traits such as jumping, sprinting, and endurance, and mentions gene-environment interactions in athletic performance.
De la Iglesia et al., 2020	The study investigates genetic influences on endurance performance and includes analysis of sport efficiency variables.
Ahmetov et al., 2016	The study examines genetic influences on specific sports-related traits, particularly endurance and power/strength-related characteristics.
La Montagna et al., 2019	The study investigates genetic influences on specific sports-related traits including performance enhancement and injury susceptibility, and considers gene-environment interactions through personalized training and nutrition programs.
Majidi et al., 2021	The study investigates specific sports-related traits including muscle mass, physical fitness, and performance in different types of athletics (sprinting vs. endurance).
Buxens et al., 2009	The study investigates genetic influences on specific sports-related traits, particularly distinguishing between endurance and power performance.
Vostrikova et al., 2022	The study investigates genetic influences on specific sports-related traits including endurance, speed, and power qualities in martial arts athletes.
McAuley et al., 2022	The study investigates genetic influences on specific sports-related traits, particularly focusing on injury risk and susceptibility, which is a crucial performance-related outcome.

Psatha et al., 2024	The study specifically investigates genetic influences on endurance and power performance traits.
Ginevičienė et al., 2021	The study investigates specific sports-related traits (muscle strength, power) and examines the genetic influence on these performance measures, linking genotypes to performance outcomes.
Monnerat et al., 2018	The study investigates multiple sports-related traits, such as hypertrophy, energy expenditure, VO2max, and recovery, examining the genetic influences on these specific performance traits.
Varillas-Delgado et al., 2022	The study investigates genetic influences on specific sports-related traits including endurance performance, cardiorespiratory fitness, energy efficiency, and muscle injury susceptibility.
Ben-Zaken et al., 2013	The study investigates genetic influences on specific sports-related traits, particularly power-speed and endurance capabilities.
Jacob et al., 2016	The study investigates specific sports-related traits, such as endurance, power, and technical skill, and examines the gene-performance relationships for these performance domains.
Onori et al., 2022	The study investigates genetic influences on injury risk, which is a key performance-related trait in sports.
Ben-Zaken et al., 2017	The study investigates the genetic influence on endurance performance and the transition from middle- to long-distance running, focusing on specific sports-related traits.
Jacob et al., 2021	The study investigates endurance performance specifically, with a clear connection between the genetic variants and the performance outcome in the sport-specific context.
Jacob et al., 2019	While specific performance traits aren't directly measured, the study investigates genetic variants known to be associated with athletic performance in elite sport, which is relevant to the performance traits criterion.
Jacob et al., 2018	The study investigates specific sports-related traits, including aerobic performance (time trials), sport-specific skills (handball, kicking), match performance metrics, and motor learning aspects.
Galeandro et al., 2020	The study investigates genetic influences on athletic performance in soccer players and includes analysis of training effects on genetic markers.
Da Rosa et al., 2022	The study investigates specific sports-related traits, including sprint ability, jumping power, and endurance, and examines the relationships between these performance traits and genetic factors.
Pickering & Kiely, 2019	The study investigates genetic influences on specific sports-related traits (endurance and speed-power capabilities).
Artells et al., 2016	The study investigates genetic influences on injury risk and recovery time, which are relevant sports-related traits.
Ebert et al., 2023	The study investigates both performance (medalist status) and injury risk factors, examining gene-performance relationships for specific sports-related traits.

Lopez-León et al., 2017	The study investigates genetic influences on endurance performance, which is a specific sports-related trait.
Pickering et al., 2021	The study specifically investigates sprint performance, including muscle fiber type analysis, and examines the relationship between genetic factors and athletic performance traits.
Gronek et al., 2020	The study investigates multiple specific sports-related traits, including endurance, power, speed, and recovery, and analyzes the gene-performance relationship.
Znazen et al., 2019	The study investigates genetic influences on specific sports-related traits, distinguishing between endurance and power performance.
Ahmetov et al., 2024	The study explicitly investigates genetic influences on sports-related traits, including endurance, power, and strength, as well as responses to training.
Moir et al., 2020	The study specifically investigates genetic influences on endurance-related traits and marathon running performance.
Petr et al., 2020	The study investigates genetic influences on specific sports-related traits including strength, power, endurance, and performance in different sports disciplines.
Bray et al., 2008	The study specifically examines genetic influences on "fitness and performance phenotypes" including responses to acute exercise and training adaptations.
Weyerstraß et al., 2017	The study investigates genetic influences on power athlete status, which is a specific sports-related trait.
Bulğay et al., 2024	The study investigates genetic influences on specific sports-related traits, differentiating between sprint/power and endurance performance.
Morucci et al., 2015	The study investigates genetic influences on specific sports-related traits, including power features and apparatus-specific performance, and analyzes clear gene-performance relationships.
Jones et al., 2018	The study investigates power and endurance qualities, as well as gene-training interactions, meeting the performance traits criterion.
Pickering et al., 2018	The study investigates aerobic fitness adaptations, examining gene-training interaction and sport-specific performance outcomes.
Meckel et al., 2020	The study investigates endurance trainability and links genetic factors to VO2 max response, which are relevant to sports-related traits.
Papadimitriou et al., 2016	The study is a primary research study with a clear methodology, statistical analysis, and a large cohort study design, indicating that it is a primary research study with empirical data.
Eynon et al., 2014	This is clearly a primary research study with empirical data, including specific methodologies for DNA extraction and genotyping.
Cieszczyk et al., 2011	This is clearly a primary research study with empirical data comparing athletes to controls.
Cięszczyk et al., 2011	This is a primary research study with empirical data, specifically a case-control study comparing athletes to controls, meeting the study design criterion.

Płoćiennik et al., 2018	This is a primary research study with empirical data, using a case-control design with clear methodology.
Grenda et al., 2014	This is clearly a primary research study with empirical data, including genetic analysis and performance comparisons.
Papadimitriou et al., 2018	The study is a primary research study with a large sample size (n=1064), clear empirical data collection, and analysis, indicating that it meets the study design criterion.
Orysiak et al., 2015	This is a primary research study with empirical data, comparing athletes to controls and analyzing genetic polymorphisms.
Domańska-Senderowska et al., 2019	This appears to be a primary research study with empirical data collection and analysis.
Eider et al., 2013	This is clearly a primary research study with empirical data collection and analysis.

Table S6. Characteristics of Included Studies for Measurable Outcomes

Author(s) & Year	Measurable Outcomes
Ahmetov et al., 2022	The abstract discusses specific genetic markers associated with quantifiable athletic performance traits (endurance, power, strength) and injury risk. It mentions 220 DNA polymorphisms with specific associations.
Massidda et al., 2014	The study includes clear quantifiable performance outcomes, specifically vertical jump performance. The study reports the specific variance explained by genetic factors, which ranges from 17.68-24.24%. The measurement approach for the performance outcome is well-defined.
Dionísio et al., 2017	The study includes quantifiable performance outcomes such as jumping, sprinting, and endurance tests, as well as measurable cardiac and hemodynamic parameters. Specific genetic polymorphisms (ACTN3, AMPD1, ACE, AGT) were also measured, indicating the study examined quantifiable sports-related phenotypes.
De la Iglesia et al., 2020	The study includes quantifiable performance outcomes, specifically VO2 maximum, VO2 VT1, and VO2 VT2, which are well-established measurable performance metrics.
Ahmetov et al., 2016	The study includes quantifiable performance outcomes and sports-related phenotypes, such as 155 genetic markers identified, 93 endurance-related, and 62 power/strength-related.
La Montagna et al., 2019	The study includes quantifiable performance outcomes such as distance covered per match, high-intensity actions per match, and injury rates. The abstract specifically mentions measuring athletic performance and injury rates.
Majidi et al., 2021	The study includes quantifiable outcomes such as BMI, body fat percentage, muscle mass, testosterone levels, and physical fitness measurements. The abstract specifically mentions measurable genetic variations (RR, RX, XX genotypes) and their relationships to performance.

Buxens et al., 2009	The study includes quantifiable performance outcomes, specifically analyzing genetic variants to discriminate between endurance and power athletes. It uses statistical measures like ROC curve analysis and multivariate logistic regression with specific performance outcomes.
Vostrikova et al., 2022	The study includes quantifiable outcomes including athletic performance measures, genetic scores (TGS), and specific genetic polymorphisms. They measure athletic ability and divide subjects into groups based on qualifications.
McAuley et al., 2022	The study examines quantifiable outcomes related to sports injuries, specifically focusing on genetic associations with various types of injuries in football players. The outcomes are clearly measurable (e.g., ACL injuries, non-contact muscle injuries).
Psatha et al., 2024	The study examines quantifiable athletic performance outcomes in power and endurance sports through meta-analysis. The abstract specifically mentions analyzing performance in endurance and power sports.
Ginevičienė et al., 2021	The study includes clear quantifiable outcomes such as grip strength and vertical jump measurements, as well as specific genetic polymorphisms (ACE I/D and ACTN3 R/X) that were measured.
Monnerat et al., 2018	The study includes clear quantifiable performance outcomes and sports-related phenotypes, such as anthropometric measurements, field tests, isokinetic tests, and physiological parameters like VO2max, hypertrophy, and energy expenditure.
Varillas-Delgado et al., 2022	The study includes quantifiable performance outcomes, such as the distribution of genetic profiles in athletes vs non-athletes, and reports specific statistical outcomes (odds ratios with confidence intervals).
Ben-Zaken et al., 2013	The study includes quantifiable performance outcomes through the use of polygenetic scores (PGDS2, PGDS5, EGDS2, EGDS5) with specific numerical values and statistical comparisons between groups.
Jacob et al., 2016	The study includes clear quantifiable performance outcomes, such as tests of endurance, power, and technical skill. The statistical analysis provides specific p-values, indicating the study used appropriate methods to measure and analyze these performance metrics.
Onori et al., 2022	The study includes quantifiable performance outcomes, specifically measuring injury incidence and analyzing statistical significance ($\chi 2$ and p-values provided for different injury types).
Ben-Zaken et al., 2017	The study includes quantifiable performance outcomes such as personal records in running, as well as specific genetic distance scores (EGDS9 and PGDS5). The statistical analysis provides p-values and an effect size of 0.75, indicating the inclusion of measurable and quantifiable data.
Jacob et al., 2021	The study includes clear quantifiable performance outcomes, specifically two-kilometre running time-trials that were measured twice, six weeks apart. The statistical analysis of these performance results is provided.
Jacob et al., 2019	The study includes quantifiable performance outcomes by examining genetic variants (ACTN3 and ACE) in relation to elite athletic status, with specific percentages and statistical

	comparisons provided.
Jacob et al., 2018	The study includes clear quantifiable performance outcomes, including 3x1km time trials, ARF-specific skill assessments (handball and kicking), and match performance metrics (DGIs/min). The statistical significance values for these outcomes are also reported.
Galeandro et al., 2020	The study includes quantifiable outcomes, specifically measuring mtDNA content in peripheral blood and analyzing specific genetic variants (ACTN3 R577X and ACE I/D polymorphisms). The outcomes are clearly measurable and quantifiable.
Da Rosa et al., 2022	The study includes quantifiable performance outcomes such as 30m sprint times, squat jump (SJ) performance, and VO2max measurements, which are specific athletic performance metrics that can be measured and compared.
Pickering & Kiely, 2019	The study examines quantifiable genetic markers (68 variants for endurance and 48 for speed-power) and their relationship to elite athlete status. The outcomes are clearly measurable, though specific performance metrics aren't detailed.
Artells et al., 2016	The study includes quantifiable performance outcomes such as injury rates, severity, and recovery time for MCL injuries. These are specific, measurable outcomes.
Ebert et al., 2023	The study includes quantifiable performance outcomes, such as medalist status, as well as specific injury outcomes with precise numbers. It also reports genetic associations with measurable odds ratios, indicating the study includes quantifiable sports-related phenotypes.
Lopez-León et al., 2017	The study includes quantifiable performance outcomes through genetic polymorphism frequencies and odds ratios in relation to endurance performance. The results are statistically analyzed with specific odds ratios and confidence intervals provided.
Pickering et al., 2021	The study includes quantifiable performance outcomes, specifically 5m sprint test performance, and identifies specific genetic variants (SNPs) associated with these performance measures. Statistical significance values are also provided, indicating the study used rigorous quantitative analysis.
Gronek et al., 2020	The study includes clear quantifiable performance outcomes or sports-related phenotypes, such as VO2max, speed (20m run), power (vertical jump), recovery (step-test), and speed endurance. The specific measurements and functional tests are described in detail.
Znazen et al., 2019	The study includes quantifiable performance outcomes, specifically examining ACE genotype distributions and their relationship to athletic performance. The study provides specific statistical results ($\chi 2$ values, P-values) and compares between groups.
Ahmetov et al., 2024	The abstract discusses "physical activity traits" and "athlete status", suggesting the study includes quantifiable performance outcomes. Additionally, the mention of studying "individual responses to training" implies the inclusion of measurable outcomes.
Moir et al., 2020	The study includes quantifiable performance outcomes related to marathon running performance, as the abstract focuses on examining genetic associations with elite marathon running performance.
Petr et al., 2020	The study examines quantifiable performance outcomes related to elite sports status, strength, power, and endurance performance, which are measurable and sports-related

	phenotypes.
Bray et al., 2008	The study includes quantifiable performance outcomes and sports-related phenotypes, as the abstract discusses "physical performance and health-related fitness phenotypes" and mentions specific measurable outcomes.
Weyerstraß et al., 2017	The study includes quantifiable performance outcomes through the examination of power athlete status, with clear measures such as odds ratios and statistical analyses.
Bulğay et al., 2024	The study includes quantifiable performance outcomes, using athletes' personal bests (PBs) rated with the World Athletics (WA) score.
Morucci et al., 2015	The study includes clear quantifiable performance outcomes in gymnastic-specific exercises, measured performances across multiple apparatus, and specific training parameters (volume, intensity, density).
Jones et al., 2018	The study includes clear quantifiable performance outcomes, including countermovement jump (CMJ) and aerobic 3-min cycle test (Aero3), with pre and post measurements and statistical analysis. Specific performance metrics are reported with p-values, indicating the study meets this criterion strongly.
Pickering et al., 2018	The study includes clear quantifiable performance outcomes, such as Yo-Yo test performance, with specific measurements of improvements (58%, 35%, 7% for different groups) and reported effect sizes (d = 2.59, d = 1.32).
Meckel et al., 2020	The study includes quantifiable performance outcomes such as VO2 max measurements and specific genetic prevalence measurements across different athlete groups, which are directly relevant to the criterion of measurable outcomes.
Papadimitriou et al., 2016	The study includes clear quantifiable performance outcomes, such as 100m, 200m, and 400m sprint times, with specific measurements provided (e.g., "21.19 ± 0.53 s vs. 21.86 ± 0.54 s"). The statistical analysis of the performance variance indicates that the study includes measurable outcomes.
Eynon et al., 2014	The study includes quantifiable genetic polymorphism (ACTN3 R577X) in relation to athletic status, with specific genotype distributions and statistical analyses (odds ratios, p-values) reported.
Cieszczyk et al., 2011	The study includes quantifiable performance outcomes, such as genotype distributions and allele frequencies with specific percentages and statistical significance (p-values).
Cięszczyk et al., 2011	The study includes quantifiable performance outcomes, specifically examining sprint and power ability in athletes. Statistical comparisons of genotype distributions (P-values) are reported, indicating the inclusion of measurable outcomes.
Płóciennik et al., 2018	The study includes quantifiable genetic polymorphisms (PEPs) with statistical analysis, including specific metrics like area under ROC curve (0.715) and epistatic effects (5.43%), indicating the inclusion of measurable performance outcomes.
Grenda et al., 2014	The study clearly examines quantifiable performance outcomes in swimming, specifically comparing sprint and endurance performance between short distance swimmers (SDS) and long distance swimmers (LDS). The outcomes are measured through genetic associations.

Papadimitriou et al., 2018	The study includes clear quantifiable performance outcomes, such as running times for 1500m, 3000m, 5000m, and marathon events. Specific numerical results with statistical analysis are provided, indicating that the study includes measurable sports-related phenotypes.
Orysiak et al., 2015	The study includes quantifiable performance outcomes, specifically examining results at 200m and 1000m distances in canoe sprint athletes. The study also provides specific odds ratios and confidence intervals.
Domańska-Senderowska et al., 2019	The abstract clearly shows quantifiable performance outcomes through jump tests (SJ and CMJ) with statistical analysis (p-values provided).
Eider et al., 2013	The study includes quantifiable performance outcomes by examining ACE genotype distributions and allele frequencies in athletes versus controls. The outcomes are concrete and measurable.

Table S7. Characteristics of Included Studies for Clinical Focus

Author(s) & Year	Clinical Focus
Ahmetov et al., 2022	The study examines genetic factors in relation to performance outcomes, going well beyond just genetic diseases. It includes performance traits, injury risk, and training responses.
Massidda et al., 2014	The study focuses on performance-related outcomes, going beyond genetic diseases to examine athletic performance variation.
Dionísio et al., 2017	The study focuses on performance-related outcomes and includes both performance and physiological parameters, rather than just genetic diseases.
De la Iglesia et al., 2020	The study focuses on performance-related outcomes rather than genetic diseases, examining genetic factors in relation to sport performance.
Ahmetov et al., 2016	The study examines genetic factors in relation to athletic performance, going beyond mere genetic diseases to look at performance-related outcomes.
La Montagna et al., 2019	The study examines genetic factors in relation to performance outcomes, going beyond just genetic diseases to look at performance enhancement and injury prevention.
Majidi et al., 2021	The study examines genetic factors (Alpha-actin3) in relation to athletic performance, going beyond mere genetic diseases to examine performance-related outcomes.
Buxens et al., 2009	The study examines genetic factors in relation to athletic performance, going beyond genetic diseases to look at performance-related outcomes.
Vostrikova et al., 2022	While the study considers cardiovascular disease risk, it primarily focuses on performance-related outcomes and athletic success, examining genetic factors beyond just diseases.
McAuley et al., 2022	The study examines genetic factors in relation to performance-related outcomes (injury risk), going beyond mere genetic diseases.

Psatha et al., 2024	The study examines genetic factors in relation to performance outcomes, going beyond merely genetic diseases to look at performance associations.
Ginevičienė et al., 2021	The study examines performance-related outcomes and focuses on athletic performance rather than disease states.
Monnerat et al., 2018	The study focuses on performance-related outcomes and genetic factors, not just genetic diseases, aligning with the clinical focus criteria.
Varillas-Delgado et al., 2022	The study examines performance-related genetic factors beyond just diseases, looking at various performance-related polymorphisms.
Ben-Zaken et al., 2013	The study examines genetic factors related to athletic performance rather than genetic diseases, focusing on performance-related polymorphisms.
Jacob et al., 2016	The study focuses on performance-related outcomes and examines genetic factors in relation to athletic ability, rather than just genetic diseases.
Onori et al., 2022	The study examines genetic factors in relation to performance and injury risk, going beyond purely genetic diseases.
Ben-Zaken et al., 2017	The study focuses on performance-related outcomes and examines genetic factors in relation to athletic success, rather than just genetic diseases.
Jacob et al., 2021	The focus of the study is on performance-related outcomes, examining genetic factors in relation to athletic performance, rather than just genetic diseases.
Jacob et al., 2019	The study examines performance-related genetic factors (ACTN3 and ACE) rather than focusing on genetic diseases, meeting the clinical focus criterion.
Jacob et al., 2018	The focus is on performance-related outcomes, with no emphasis on genetic diseases. There is a clear connection to athletic performance metrics.
Galeandro et al., 2020	The study examines genetic factors in relation to athletic performance, going beyond merely genetic diseases.
Da Rosa et al., 2022	The study focuses on performance-related outcomes and examines genetic factors in relation to athletic performance, not diseases.
Pickering & Kiely, 2019	The study focuses on athletic performance and genetic factors beyond just diseases, examining genetic profiles related to athletic capability.
Artells et al., 2016	The study examines genetic factors in relation to performance and injury outcomes, going beyond mere genetic diseases.
Ebert et al., 2023	The study focuses on both performance and injury-related outcomes, going beyond just genetic diseases to examine athletic performance factors.
Lopez-León et al., 2017	The study examines genetic factors in relation to athletic performance rather than disease, focusing on the PPARA gene's role in endurance ability.
Pickering et al., 2021	The study examines genetic factors related to athletic performance, specifically sprint speed, rather than just genetic diseases, indicating a focus on performance-related outcomes.

Gronek et al., 2020	The study examines performance-related outcomes, going beyond genetic diseases to look at athletic performance markers.
Znazen et al., 2019	The study examines genetic factors in relation to athletic performance, going beyond mere genetic diseases to look at performance-related outcomes.
Ahmetov et al., 2024	The study examines genetic factors in relation to performance outcomes, going beyond just genetic diseases to include athletic performance and training responses.
Moir et al., 2020	The study examines genetic factors in relation to performance outcomes, going beyond just genetic diseases to look at performance-related genetic associations.
Petr et al., 2020	The study examines genetic factors (PPARs) in relation to performance outcomes, going beyond mere genetic diseases.
Bray et al., 2008	The study focuses on performance-related outcomes and fitness phenotypes, going beyond just genetic diseases.
Weyerstraß et al., 2017	The study examines genetic polymorphisms in relation to athletic performance, going beyond mere genetic diseases to look at performance-related outcomes.
Bulğay et al., 2024	The study examines genetic factors in relation to athletic performance, going beyond mere genetic diseases.
Morucci et al., 2015	The study examines performance-related genetic factors, with a focus on athletic performance rather than genetic diseases.
Jones et al., 2018	The study focuses on performance-related outcomes and examines genetic factors in relation to training response, rather than just genetic diseases, meeting the clinical focus criterion.
Pickering et al., 2018	The focus is on performance-related outcomes, examining genetic factors in relation to training adaptation, rather than being disease-focused.
Meckel et al., 2020	The study examines performance-related outcomes, going beyond just genetic diseases to look at athletic performance implications.
Papadimitriou et al., 2016	The study examines genetic factors in relation to athletic performance, rather than just genetic diseases, indicating a clinical focus on performance-related outcomes.
Eynon et al., 2014	The study examines genetic factors (ACTN3 polymorphism) in relation to athletic performance, going beyond mere genetic diseases.
Cieszczyk et al., 2011	The study examines genetic factors in relation to athletic performance, going beyond mere genetic diseases.
Cięszczyk et al., 2011	The study focuses on performance-related outcomes (sprint and power ability) rather than just genetic diseases, meeting the clinical focus criterion.
Płoćiennik et al., 2018	The study focuses on performance-related outcomes and genetic factors related to athletic ability, not genetic diseases.
Grenda et al., 2014	The study examines performance-related genetic factors (ACE I/D and ACTN3 R577X polymorphisms) rather than focusing on genetic diseases.

Papadimitriou et al., 2018	The study focuses on performance-related outcomes and examines genetic factors in relation to athletic performance, rather than just genetic diseases, indicating a clinical focus beyond just genetic diseases.
Orysiak et al., 2015	The study examines genetic factors (ACTN3 R577X polymorphism) in relation to athletic performance, going beyond mere genetic diseases.
Domańska-Senderowska et al., 2019	The study focuses on performance-related outcomes (jump performance) rather than genetic diseases.
Eider et al., 2013	The study examines genetic factors (ACE gene) in relation to athletic performance, going beyond mere disease associations.

Table S8. Characteristics of Included Studies for effect size

Author(s) & Year	Effect size
Ahmetov et al., 2022	While the abstract discusses associations between genetic markers and outcomes, it doesn't explicitly mention effect sizes. However, it does reference meta-analyses which typically include effect sizes.
Massidda et al., 2014	The study reports the explained variance (17.68-24.24%), which quantifies the genetic contribution to performance.
Dionísio et al., 2017	While the study reports associations between genotypes and performance, the abstract does not explicitly state the effect sizes. The results are described qualitatively as "better performance" and "better results".
De la Iglesia et al., 2020	While statistical significance is mentioned (P-values), the abstract does not explicitly state effect sizes. However, relationships between variables are quantified.
Ahmetov et al., 2016	While the abstract mentions associations between genetic markers and athlete status, specific effect sizes are not explicitly stated. However, it does note that some markers were replicated across multiple studies.
La Montagna et al., 2019	While the abstract mentions finding "significantly enriched" polymorphisms, it doesn't provide specific effect sizes or quantitative measures of the associations.
Majidi et al., 2021	While the abstract mentions "significant" differences and relationships, specific effect sizes are not explicitly stated. However, it does mention that certain genetic variants were found in "less than 1%" of exceled athletes.
Buxens et al., 2009	The abstract reports specific effect measures, including the contribution of genetic factors (21.4%) and ROC curve area (0.72, CI: 0.66-0.81).
Vostrikova et al., 2022	While the study examines associations between genetic variants and performance, specific effect sizes are not explicitly mentioned in the abstract. However, they do report "significant differences" between groups.
McAuley et al.,	While the abstract mentions associations, it doesn't explicitly report effect sizes. However, as

2022	this is a synthesis of multiple studies, effect sizes were likely considered in the full text.
Psatha et al., 2024	The meta-analysis nature of the study implies effect size analysis, though specific effect sizes aren't detailed in the abstract. The study mentions "statistically significant association" analysis.
Ginevičienė et al., 2021	While comparative results are mentioned ("better in athletes with ACE I/I"), specific effect sizes are not detailed in the abstract.
Monnerat et al., 2018	While statistical significance is mentioned (P < .00001), specific effect sizes are not directly reported in the abstract, so the information is partial or unclear.
Varillas-Delgado et al., 2022	The study reports odds ratios (OR) with confidence intervals, providing clear effect size measurements for different genetic profiles.
Ben-Zaken et al., 2013	While statistical significance (p-values) is reported, explicit effect sizes are not mentioned in the abstract. However, mean differences between groups are provided.
Jacob et al., 2016	While the study reports statistical significance through p-values, the actual effect sizes are not explicitly mentioned in the abstract.
Onori et al., 2022	While statistical significance is reported (p-values), the abstract doesn't explicitly mention effect sizes. However, chi-square values are provided which can be used to calculate effect sizes.
Ben-Zaken et al., 2017	The effect size of 0.75 is explicitly reported in the study.
Jacob et al., 2021	The statistical significance of the results is reported through p-values, but the actual effect sizes are not explicitly stated in the abstract.
Jacob et al., 2019	While not explicitly stated in traditional effect size measures, the study provides odds ratios and clear statistical comparisons (e.g., 4.3% vs 28.8% for XX genotype, $\chi 2$ values) that can be used to infer the effect size.
Jacob et al., 2018	While statistical significance (p-values) is reported, the effect sizes are not explicitly mentioned in the abstract.
Galeandro et al., 2020	While the study examines associations, the abstract doesn't explicitly report effect sizes. However, this information might be available in the full text.
Da Rosa et al., 2022	While comparisons between groups are made, specific effect sizes are not explicitly reported in the abstract. The results are described qualitatively (e.g., "bigger," "higher," "lower").
Pickering & Kiely, 2019	While the study compares groups, specific effect sizes aren't reported in the abstract. This creates some uncertainty about the strength of associations found.
Artells et al., 2016	While the abstract mentions differences between genotypes (16 MCL injuries for ELN-AA vs. 3 for ELN-AG vs. 0 for ELN-GG), precise effect sizes are not reported. However, the relationships appear meaningful.
Ebert et al., 2023	The study clearly reports effect sizes through odds ratios, including notable effects such as OR 14.3 for DSG1 and OR 17.4 for DSG4.

Lopez-León et al., 2017	Effect sizes are clearly reported through odds ratios (e.g., GG vs CC genotype OR=2.37, 95% CI 1.40-3.99).
Pickering et al., 2021	While explicit effect sizes are not mentioned in the abstract, the study design suggests that effect sizes were likely calculated, as the abstract reports statistical significance (p-values).
Gronek et al., 2020	While the results are mentioned, the specific effect sizes are not explicitly stated in the abstract. The abstract only mentions "tendencies" and non-significant findings.
Znazen et al., 2019	While statistical significance is reported, explicit effect sizes are not mentioned in the abstract, creating some uncertainty, though the underlying data likely contains this information.
Ahmetov et al., 2024	While the abstract does not explicitly discuss effect sizes, Elicit notes that as a review of genomic studies, effect sizes are likely included in the full text.
Moir et al., 2020	While the abstract does not explicitly state the effect sizes, Elicit notes that as a systematic review, the full text likely contains this information.
Petr et al., 2020	While the study examines associations between genetic variants and athletic performance, specific effect sizes are not explicitly mentioned in the abstract. However, as this is a systematic review, effect sizes may be included in the full text.
Bray et al., 2008	While the abstract discusses associations between genes and performance, it notes that "most studies reported to date are based on small sample sizes," indicating a lack of specific effect size information in the abstract.
Weyerstraß et al., 2017	The study reports odds ratios (ORs) with 95% confidence intervals, providing clear effect size measurements for the genetic associations.
Bulğay et al., 2024	While statistical significance is mentioned ($p < 0.05$), specific effect sizes are not detailed in the abstract.
Morucci et al., 2015	While statistical significance is mentioned, specific effect sizes are not explicitly stated in the abstract. The study uses terms like "significantly lower" but does not quantify the difference.
Jones et al., 2018	While the abstract does not explicitly report effect sizes, it does state that the study compares performance changes between groups, suggesting that some measure of effect size is likely available in the full paper.
Pickering et al., 2018	The study reports Cohen's d effect sizes between groups, with large effect sizes documented ($d = 2.59$, $d = 1.32$).
Meckel et al., 2020	Elicit mentions that the polymorphism explains 6.1% of VO2 max response variance, but notes that this is referenced from previous work, not the current study.
Papadimitriou et al., 2016	The study reports specific effect sizes, stating that the ACTN3 and ACE variants account for 0.92% and 1.48% of sprint time variance, respectively, and provides concrete time differences between genotype groups.
Eynon et al., 2014	The abstract reports effect sizes in terms of odds ratios (0.58, 95% CI: 0.34-0.39).
Cieszczyk et al.,	While statistical significance is reported, explicit effect sizes are not mentioned in the

2011	abstract. However, the relative frequencies between groups provide some indication of effect magnitude.
Cięszczyk et al., 2011	While statistical significance is reported, the abstract does not explicitly state effect sizes. However, the study reports allele frequencies (30.69% vs. 40.35%), which provides some indication of the magnitude of difference.
Płóciennik et al., 2018	The study reports specific effect sizes, including the synergistic effect of 5.43% and provides statistical measures of association.
Grenda et al., 2014	While the study discusses associations and statistical significance, it doesn't explicitly mention effect sizes in the abstract. However, this information might be available in the full text.
Papadimitriou et al., 2018	The study reports specific performance differences between genotype groups and includes statistical significance values and standard deviations, indicating that it examines effect size.
Orysiak et al., 2015	Effect sizes are reported through odds ratios (OR=2.95, 95% CI: 1.37-6.35 for 1000m athletes
Domańska-Senderowska et al., 2019	While statistical significance is reported (p-values), no effect sizes are explicitly mentioned.
Eider et al., 2013	While not explicitly stated in traditional effect size metrics, the study reports clear quantitative differences in allele frequencies between groups (63.0% vs 53.2% for D allele) and shows a dose relationship across performance levels.

Table S9. Characteristics of Included Studies for Screening judgement

Author(s) & Year	Screening judgement
Ahmetov et al., 2022	This comprehensive sports genomics study thoroughly examines genetic markers associated with athletic performance across multiple domains including endurance, power, and strength, while identifying specific DNA polymorphisms linked to performance traits and injury risk. The research systematically analyzes human athletic populations, investigating genetic variations through advanced methodologies like whole genome sequencing and genome-wide association studies, with a robust focus on quantifiable performance-related genetic factors. By exploring 220 DNA polymorphisms and their relationships to athletic phenotypes, the study provides a sophisticated genetic perspective on sports performance and physiological potential.
Massidda et al., 2014	This study comprehensively examines genetic polymorphisms in top-level male soccer players, demonstrating a robust investigation of performance-related genetic variations with quantifiable outcomes in vertical jump performance. The research meets all screening

	criteria by providing empirical data on specific genetic factors influencing athletic performance, including variance explanation and potential implications for individualized training and injury prevention.
Dionísio et al., 2017	A comprehensive genetic study of 220 male soccer athletes examining multiple polymorphisms and their relationship to performance traits like jumping, sprinting, and endurance. The research systematically investigated genetic variations across different genotypes and their potential impacts on athletic performance, utilizing quantifiable physiological and performance measurements. Specific genetic factors were analyzed in relation to sports-related phenotypes, providing insights into potential genetic influences on athletic capabilities.
De la Iglesia et al., 2020	This cross-sectional study on male cyclists comprehensively examines genetic influences on endurance performance through a genetic prediction score, utilizing quantifiable physiological metrics like VO2 measurements. The research robustly investigates genetic polymorphisms' relationship to athletic performance, demonstrating statistically significant associations between genetic factors and sports-related phenotypes across multiple performance variables.
Ahmetov et al., 2016	This comprehensive review systematically explores genetic determinants of athletic performance across multiple human populations, identifying 155 genetic markers associated with endurance and power-related athletic traits. The study rigorously examines genetic polymorphisms through genome-wide association studies, providing quantifiable insights into the genetic foundations of elite athletic status across diverse ethnic groups. By investigating performance-related genetic variations beyond disease markers, the research offers a nuanced understanding of the genetic mechanisms underlying athletic potential.
La Montagna et al., 2019	A comprehensive genetic study of professional soccer players examined genetic polymorphisms across five key genes to understand performance enhancement and injury prevention. The research successfully integrated genetic screening with quantifiable athletic outcomes, demonstrating significant potential for personalized training and nutritional interventions based on individual genetic profiles. By analyzing genetic variations in relation to sports performance, the study provides a multidisciplinary approach to understanding athlete-specific genetic predispositions.
Majidi et al., 2021	This study comprehensively examines the Alpha-actin3 gene's role in athletic performance, demonstrating strong genetic influences on muscle mass, testosterone levels, and sport-specific traits across different athlete genotypes. By analyzing quantifiable outcomes including genotype distributions, physical fitness, and performance characteristics, the research provides nuanced insights into genetic variations' impact on athletic potential and performance differentiation.
Buxens et al., 2009	This comprehensive genetic study of world-class athletes systematically examined genetic variants across multiple performance domains, utilizing robust statistical methods to quantify the genetic contribution to athletic performance. By analyzing 36 genetic variants in endurance and power athletes, the research provides nuanced insights into the complex interplay of genetic factors underlying elite sports performance, demonstrating a sophisticated approach to understanding athletic potential beyond simple genetic determinism.

Vostrikova et al., 2022	A comprehensive genetic study of martial arts athletes examining polymorphic loci across four genes reveals significant associations between genetic variants and athletic performance traits like endurance and power qualities. The research systematically investigates genetic predispositions to athletic success through quantifiable genetic scoring and performance categorization, demonstrating a robust approach to understanding genetic influences on sports performance.
McAuley et al., 2022	A comprehensive genetic study of football players examining injury risk through multiple genetic polymorphisms, involving 9,642 human participants across 34 studies. The research systematically investigates genetic factors related to sports performance, specifically focusing on injury susceptibility across multiple genes and demonstrating a robust methodological approach to understanding genetic contributions to athletic traits. Despite some methodological limitations, the study provides significant insights into genetic associations with sports-related injury risks.
Psatha et al., 2024	A comprehensive meta-analysis examining genetic variants across 4,228 articles revealed no statistically significant association between genomic factors and athletic performance in endurance and power sports. The study involved 54,382 human subjects, including 11,501 athletes, and systematically analyzed 37 different genes with a focus on performance-related genetic influences. Rigorous methodology and large sample size provide robust evidence challenging genetic performance predictions.
Ginevičienė et al., 2021	This study comprehensively examines genetic polymorphisms in Lithuanian athletes, measuring quantifiable performance outcomes like grip strength and vertical jump across a human subject population. The research systematically investigates genetic factors (ACE and ACTN3 genes) in relation to athletic performance, providing empirical data on how specific genetic variations potentially influence sports-related physical traits. By including both elite athletes and a control group, the study offers robust insights into the genetic determinants of athletic performance.
Monnerat et al., 2018	This study of professional soccer players comprehensively examines genetic polymorphisms and performance-related traits through robust quantitative measurements and genetic analysis. The research meets critical screening criteria by focusing on human athletes, investigating performance-related genetic factors, and utilizing empirical data collection across multiple physiological and genetic parameters. Genetic influences on sports performance are explored through sophisticated methodological approaches including population genetics and genomic database comparisons.
Varillas-Delgado et al., 2022	This comprehensive genetic study thoroughly examines performance-related polymorphisms across multiple physiological domains in professional athletes, comparing genetic distributions between athletes and non-athletes. The research systematically investigates genetic factors influencing endurance, cardiorespiratory fitness, energy efficiency, and muscle injury susceptibility through quantitative analysis of 452 human subjects, providing robust statistical evidence of genetic variations associated with athletic performance.
Ben-Zaken et al., 2013	This comprehensive genetic study explores performance-related polymorphisms across power-speed and endurance athletes, utilizing quantifiable polygenetic scores and comparing multiple genetic variations. The research systematically examines human subjects across three distinct groups, providing statistically significant insights into genetic

	factors influencing athletic performance through detailed genetic polymorphism analysis. Multiple genetic markers were investigated to differentiate athletic capabilities, demonstrating a robust approach to understanding genetic contributions to sports performance.
Jacob et al., 2016	This study comprehensively examines genetic polymorphisms in sub-elite Australian Football players, measuring quantifiable performance outcomes across endurance, power, and technical skill domains. By investigating specific gene variations and their relationship to athletic performance, the research provides novel insights into genetic predispositions for multidimensional sports performance. The methodology demonstrates a robust approach to understanding genetic influences on athletic traits through empirical data and statistical analysis.
Onori et al., 2022	This study comprehensively examines genetic factors influencing athletic performance and injury risk among rugby players, utilizing a robust research design with human subjects and multiple genetic markers. The research provides quantifiable outcomes related to specific gene variations and their association with total and bone injuries, demonstrating a nuanced approach to understanding genetic contributions to sports performance. By investigating gene-performance interactions across multiple genetic factors, the study offers valuable insights into personalized athletic training and injury prevention strategies.
Ben-Zaken et al., 2017	This study comprehensively examines genetic factors influencing athletic performance, specifically focusing on the transition from middle- to long-distance running among elite athletes. By analyzing genetic polymorphisms and their relationship to performance outcomes, the research provides quantifiable evidence of how genetic profiles may predict athletic success and specialization. The investigation meets multiple screening criteria through its robust methodology, human subject design, and performance-related genetic analysis.
Jacob et al., 2021	This study comprehensively examines genetic polymorphisms in relation to endurance performance among professional athletes, utilizing a robust experimental design with quantifiable outcomes across multiple genetic variants. The research focuses on human subjects from an elite athletic population, investigating specific performance-related genetic factors through empirical data collection and statistical analysis. By exploring gene-performance interactions in Australian Football players, the study provides significant insights into the genetic underpinnings of athletic endurance.
Jacob et al., 2019	This study comprehensively examines genetic variants in elite Australian Football League players, demonstrating a robust investigation of performance-related genetic factors across multiple screening criteria. By analyzing ACTN3 and ACE gene polymorphisms in human subjects with quantifiable outcomes, the research provides significant insights into genetic influences on athletic performance at the elite level.
Jacob et al., 2018	This study comprehensively examines genetic polymorphisms in sub-elite Australian Rules Football players, investigating their potential influence on athletic performance through quantifiable metrics like time trials, skill assessments, and match performance. The research systematically explores multiple genetic variants' relationships with sports-specific traits, providing empirical data on how genetic factors may contribute to athletic performance and motor learning capabilities.

Galeandro et al., 2020	This study comprehensively examines genetic factors influencing athletic performance through analysis of ACTN3 and ACE polymorphisms in soccer players, utilizing a human subject design with quantifiable genetic and performance-related outcomes. The research provides novel insights into genetic variations, mitochondrial DNA content, and potential predictors of athletic talent, meeting multiple rigorous screening criteria for sports genetics research.
Da Rosa et al., 2022	A comprehensive study of young track and field athletes examining genetic variants across sprint, jump, and endurance disciplines, with robust quantitative measurements of performance traits and genetic analysis. The research systematically investigates ACE and ACTN3 gene influences on athletic performance, utilizing a well-defined human subject population and multiple performance metrics. Genetic profiling demonstrates potential for identifying athletic talent by linking specific gene variants to performance characteristics across different athletic domains.
Pickering & Kiely, 2019	A comprehensive genetic study of elite athletes examined multiple genetic variants across endurance and speed-power performance domains, involving human subjects and focusing on performance-related genetic factors. The research compared genetic profiles between elite athletes and non-athletic controls, investigating potential genetic markers for athletic performance while demonstrating the complexity of genetic influences on athletic capability. Despite investigating multiple genetic variants, the study ultimately concluded that current genetic information cannot accurately discriminate athletic potential.
Artells et al., 2016	This study of top-class football players comprehensively examines genetic polymorphisms in the Elastin gene, revealing significant relationships between specific genotypes and medial collateral ligament (MCL) injury rates, severity, and recovery time. By investigating genetic factors directly linked to sports performance and injury risk, the research provides a novel approach to understanding individual athletic predisposition and potential injury management strategies.
Ebert et al., 2023	A comprehensive genome-wide association study of 126 Estonian National Team athletes examined genetic variants related to performance and musculoskeletal injury, identifying multiple single-nucleotide polymorphisms with significant associations to athletic performance and injury risk. The research provides quantifiable outcomes across multiple genetic factors, demonstrating strong relationships between specific genetic markers and sports-related phenotypes in an elite athletic population.
Lopez-León et al., 2017	A comprehensive meta-analysis examining genetic factors in endurance athletes reveals significant associations between the PPARA gene polymorphism and athletic performance. The study systematically analyzed 760 athletes and 1,792 controls, demonstrating quantifiable genetic variations linked to endurance capabilities through robust statistical methods. Genetic polymorphisms were found to correlate with enhanced athletic performance, providing insights into the potential genetic underpinnings of sports excellence.
Pickering et al., 2021	A genome-wide association study examining genetic variants in elite youth football players demonstrates strong alignment with performance-related genetic research criteria. The study comprehensively investigates sprint performance through multiple human cohorts, analyzing specific genetic polymorphisms associated with athletic traits across diverse populations. Rigorous genetic analysis of sports performance provides empirical insights

	into the molecular mechanisms underlying athletic capabilities.
Gronek et al., 2020	This study of elite male field hockey players comprehensively examines genetic factors related to athletic performance through quantifiable outcomes like VO2max, speed, power, and recovery. The research employs robust genetic testing and performance measurements, focusing on ACE I/D polymorphism across multiple sports-related traits while utilizing a primary research design with human subjects. Despite non-significant findings, the study provides valuable insights into genetic influences on athletic performance characteristics.
Znazen et al., 2019	A comprehensive genetic study examining ACE polymorphism in Tunisian athletes reveals significant associations between specific gene variants and athletic performance across endurance and power sports. The research systematically investigates genetic factors influencing sports performance through quantitative analysis of human subjects, demonstrating how genetic background can predispose individuals to specific athletic capabilities. By exploring gene-performance interactions, the study provides insights into the genetic foundations of athletic potential.
Ahmetov et al., 2024	This systematic review comprehensively examines genetic variants associated with physical activity and athletic performance, covering 149 variants related to physical activity traits and 253 variants linked to athlete status across endurance, power, and strength domains. The study meets key screening criteria by focusing on human subjects, genetic factors, and performance-related outcomes, providing empirical data on the genetic influences of athletic performance and training responses.
Moir et al., 2020	A comprehensive systematic review examining genetic factors in marathon running performance, involving 10,442 human participants and analyzing 160 polymorphisms across 27 genes. The study rigorously investigates performance-related genetic associations through multiple research designs, focusing on endurance traits and athletic genomics while highlighting the complexity of predicting marathon success through genetic markers.
Petr et al., 2020	This systematic review comprehensively examines genetic variations in PPARs across elite athletes, demonstrating strong associations between specific gene alleles and performance in strength, power, and endurance sports. The study rigorously investigates human genetic factors related to athletic performance, providing nuanced insights into how specific genetic markers might influence elite sports status across multiple disciplines.
Bray et al., 2008	A comprehensive genetic mapping study exploring human performance and fitness phenotypes across active and sedentary populations, examining genetic variations and their potential influences on exercise responses and training adaptations. The research systematically reviews genetic factors related to performance traits, covering 214 autosomal gene entries and quantitative trait loci, while acknowledging limitations in sample sizes and potential undiscovered gene-exercise interactions. The study provides a nuanced exploration of genetic contributions to human physical performance, bridging genetic research with exercise science.
Weyerstraß et al., 2017	A comprehensive meta-analysis examining genetic polymorphisms in power athletes, involving a large human sample of 5,834 athletes and 14,018 controls, systematically investigated genetic variations associated with athletic performance. The study rigorously analyzed multiple genetic factors across various genes, providing quantifiable outcomes through odds ratios and confidence intervals, while exploring potential performance-related

	genetic influences. By addressing genetic associations with power athlete status and employing robust statistical methodologies, the research offers significant insights into the genetic underpinnings of athletic performance.
Bulğay et al., 2024	This study comprehensively examines genetic factors influencing athletic performance through a robust investigation of the rs17602729 polymorphism across elite athletes from different sporting disciplines. By utilizing whole exome sequencing, quantifiable performance metrics, and a diverse human subject population, the research provides a nuanced exploration of potential genetic contributions to athletic performance. The methodology demonstrates strong scientific rigor in analyzing genetic variations across sprint/power and endurance athletic groups.
Morucci et al., 2015	This study comprehensively examines genetic polymorphisms in elite male gymnasts, providing quantifiable performance outcomes across multiple athletic apparatus while investigating the relationship between specific gene variations and sports performance. The research meets rigorous screening criteria by focusing on human subjects, employing a primary research design, and analyzing genetic factors directly linked to athletic traits such as power and performance potential. Genetic markers like ACE and ACTN3 were systematically studied to demonstrate potential implications for customized athletic training programs.
Jones et al., 2018	This study comprehensively examines genetic influences on athletic performance through a robust intervention design involving male athletes from multiple sports, utilizing 15 gene polymorphisms to predict and optimize resistance training responses. By measuring quantifiable performance outcomes like countermovement jump and aerobic cycle tests, the research demonstrates significant correlations between individual genetic profiles and training effectiveness, with clear statistical validation across two independent cohorts.
Pickering et al., 2018	A comprehensive study of youth soccer players examining genetic influences on aerobic training adaptations, utilizing genetic polymorphisms to predict performance improvements across different genotype groups. The research quantitatively assessed athletic performance through Yo-Yo tests, demonstrating significant variations in fitness adaptation based on genetic profiles, with clear effect sizes and empirical measurements of training outcomes. This investigation provides insights into individualized training design by linking specific genetic markers to sports performance potential.
Meckel et al., 2020	This study comprehensively examines the ACSL A/G genetic polymorphism across different athletic populations, providing quantifiable insights into genetic influences on endurance trainability and sports performance. By analyzing 167 male athletes and 60 controls, the research offers a robust investigation of genetic factors related to athletic phenotypes, with specific focus on how genetic variations might impact team selection and training strategies.
Papadimitriou et al., 2016	A comprehensive genetic study of elite sprinters examining ACTN3 and ACE gene variants reveals quantifiable performance impacts across multiple sprint distances. The research provides robust evidence of genetic influences on athletic performance, analyzing 346 athletes across multiple countries and demonstrating how specific genetic variations can account for performance variance. Precise measurements and statistical analysis offer insights into the genetic components underlying elite sprint performance.

Eynon et al., 2014	A comprehensive genetic study examining the ACTN3 R577X polymorphism across multiple European athletic cohorts, investigating potential genetic influences on team-sport, endurance, and sprint/power athletic performance. The research systematically analyzed genetic variations in human subjects, reporting quantifiable outcomes with statistical effect sizes and exploring gene-performance relationships across different athletic categories. Robust methodology and clear genetic focus provide insights into potential genetic determinants of athletic performance.
Cieszczyk et al., 2011	A comprehensive genetic study of Polish rowers examining the ACTN3 gene polymorphism revealed significant associations between specific genotypes and athletic performance. The research quantitatively analyzed genetic variations across elite and non-elite athletes, demonstrating robust methodological rigor in exploring gene-performance relationships through empirical human subject data. Findings challenge simplistic genetic performance predictions, highlighting the complex interplay between genetic markers and athletic capability.
Cięszczyk et al., 2011	A comprehensive case-control study examining the ACTN3 R577X polymorphism in athletes demonstrates strong genetic influences on sprint and power performance. The research systematically compares genotype frequencies between power-oriented athletes and non-athletes, revealing statistically significant differences in genetic markers related to athletic performance. Genetic variations were explored with rigorous methodology, focusing on specific performance-related traits across human subjects.
Płóciennik et al., 2018	A comprehensive genetic study examining performance-enhancing polymorphisms in gymnasts, involving human subjects across elite, sub-elite, and sedentary populations. The research systematically investigates genetic factors influencing athletic performance through detailed molecular analysis, quantifiable outcomes, and statistical modeling of gene interactions. Robust methodology and focus on performance-related genetic variations demonstrate a sophisticated approach to understanding the genetic basis of athletic aptitude.
Grenda et al., 2014	This study comprehensively examines genetic polymorphisms (ACE I/D and ACTN3 R577X) in relation to swimming performance, involving human subjects and focusing on elite athletes across different swimming disciplines. The research provides a detailed genetic analysis of performance-related traits, specifically investigating how specific gene variations might influence sprint and endurance capabilities in swimmers. By exploring genetic factors beyond disease states and utilizing empirical data, the study offers insights into the potential genetic foundations of athletic performance.
Papadimitriou et al., 2018	A comprehensive genetic study of endurance runners examining ACTN3 and ACE variants across multiple performance distances, involving 698 athletes from six countries. The research provides quantifiable performance outcomes, analyzes genetic factors directly related to athletic performance, and systematically investigates potential genetic influences on running times across different distances. Robust methodology includes large sample size, multiple genotype analyses, and statistical comparisons of running performances.
Orysiak et al., 2015	The study examines genetic factors (ACTN3 R577X polymorphism) in relation to athletic performance, going beyond mere genetic disases
Domańska-	A comprehensive study examining ACTN3 gene expression and athletic performance

Senderowska et al., 2019	through quantifiable jump tests involving human athletes, demonstrating significant statistical relationships between genetic factors and performance-related phenotypes. The research successfully explores genetic influences on explosive strength, providing insights into the complex interactions between genetic variations and athletic performance traits.
Eider et al., 2013	This study comprehensively examines the ACE gene polymorphism in elite Polish power athletes, demonstrating a robust investigation of genetic factors influencing athletic performance through quantifiable genetic and phenotypic measurements. The research meets all screening criteria by analyzing human subjects, providing clear genetic and performance-related outcomes, and employing a primary research design that explores gene-performance interactions across different athletic performance levels.

Table S10. GRADE Summary Statement for Results Section

Outcome	(ES)	CI Lower	CI Upper	Certainty	Risk of Bias	Inconsistency	Indirectness	Imprecision	Publication Bias
ACTN3- Power Sports	1,4	1,18	1,65	Moderate	Some concerns	High	No	No	Yes
ACTN3 - Endurance Sports	1,35	1,12	1,58	Moderate	Some concerns	Moderate	No	No	Yes
ACE -Power Sports	1,2	1,03	1,43	Low	High	High	No	Yes	Yes
ACE - Endurance Sports	1,22	1,05	1,41	Low	Moderate	Moderate	No	Yes	Yes
PPARGC1A - Endurance	1,18	1,03	1,37	Low	Moderate	Moderate	No	No	Unclear
BDNF - Power	1,21	1,08	1,39	Moderate	Some concerns	Low	No	No	Unclear
COL5A1 - Injury/Tendon	1,15	1,02	1,31	Low	Moderate	Moderate	No	Yes	Unclear
VEGF- Endurance	1,2	1,05	1,38	Low	Moderate	Low	No	No	Unclear
AMPD1 - Power Recovery	1,19	1,05	1,36	Low	Moderate	Moderate	No	No	Unclear
